# Immunogenicity and Safety of Heterologous Omicron BA.1 and Bivalent SARS-CoV-2 Recombinant Spike Protein Booster Vaccines: A Phase 3 Randomized Clinical Trial

**DOI:** 10.1093/infdis/jiad508

**Published:** 2023-11-16

**Authors:** Chijioke Bennett, E Joy Rivers, Wayne Woo, Mark Bloch, King Cheung, Paul Griffin, Rahul Mohan, Sachin Deshmukh, Mark Arya, Oscar Cumming, A Munro Neville, Toni McCallum Pardey, Joyce S Plested, Shane Cloney-Clark, Mingzhu Zhu, Raj Kalkeri, Nita Patel, Agi Buchanan, Alex Marcheschi, Jennifer Swan, Gale Smith, Iksung Cho, Gregory M Glenn, Robert Walker, Raburn M Mallory

**Affiliations:** Novavax, Inc., Gaithersburg, Maryland, USA; Novavax, Inc., Gaithersburg, Maryland, USA; Novavax, Inc., Gaithersburg, Maryland, USA; Holdsworth House Medical Practice and Kirby Institute, University of New South Wales, Sydney, New South Wales, Australia; Emeritus Research, Camberwell, Victoria, Australia; Mater Misericordiae Ltd and University of Queensland, South Brisbane, Queensland, Australia; Paratus Clinical Research Western Sydney, Blacktown, New South Wales, Australia; Griffith University, Southport, Queensland, Australia; Australian Clinical Research Network, Maroubra, New South Wales, Australia; Novatrials, Newcastle, New South Wales, Australia; AusTrials, Brisbane, Queensland, Australia; Novatrials, Newcastle, New South Wales, Australia; Novavax, Inc., Gaithersburg, Maryland, USA; Novavax, Inc., Gaithersburg, Maryland, USA; Novavax, Inc., Gaithersburg, Maryland, USA; Novavax, Inc., Gaithersburg, Maryland, USA; Novavax, Inc., Gaithersburg, Maryland, USA; Novavax, Inc., Gaithersburg, Maryland, USA; Novavax, Inc., Gaithersburg, Maryland, USA; Novavax, Inc., Gaithersburg, Maryland, USA; Novavax, Inc., Gaithersburg, Maryland, USA; Novavax, Inc., Gaithersburg, Maryland, USA; Novavax, Inc., Gaithersburg, Maryland, USA; Novavax, Inc., Gaithersburg, Maryland, USA; Novavax, Inc., Gaithersburg, Maryland, USA

**Keywords:** neutralizing antibody, NVX-CoV2373, omicron BA.1, reactogenicity, SARS-CoV-2

## Abstract

**Background:**

Mutations present in emerging SARS-CoV-2 variants permit evasion of neutralization with prototype vaccines. A novel Omicron BA.1 subvariant–specific vaccine (NVX-CoV2515) was tested alone or as a bivalent preparation with the prototype vaccine (NVX-CoV2373) to assess antibody responses to SARS-CoV-2.

**Methods:**

Participants aged 18 to 64 years immunized with 3 doses of prototype mRNA vaccines were randomized 1:1:1 to receive a single dose of NVX-CoV2515, NVX-CoV2373, or the bivalent mixture in a phase 3 study investigating heterologous boosting with SARS-CoV-2 recombinant spike protein vaccines. Immunogenicity was measured 14 and 28 days after vaccination for the SARS-CoV-2 Omicron BA.1 sublineage and ancestral strain. Safety profiles of vaccines were assessed.

**Results:**

Of participants who received trial vaccine (N = 829), those administered NVX-CoV2515 (n = 286) demonstrated a superior neutralizing antibody response to BA.1 vs NVX-CoV2373 (n = 274) at day 14 (geometric mean titer ratio, 1.6; 95% CI, 1.33–2.03). Seroresponse rates were 73.4% (91/124; 95% CI, 64.7–80.9) for NVX-CoV2515 vs 50.9% (59/116; 95% CI, 41.4–60.3) for NVX-CoV2373. All formulations were similarly well tolerated.

**Conclusions:**

NVX-CoV2515 elicited a superior neutralizing antibody response against the Omicron BA.1 subvariant as compared with NVX-CoV2373 when administered as a fourth dose. Safety data were consistent with the established safety profile of NVX-CoV2373.

**Clinical Trials Registration:**

ClinicalTrials.gov (NCT05372588).

## Teaser

The Omicron BA.1 variant–specific vaccine NVX-CoV2515 elicited a superior neutralizing antibody response against the Omicron BA.1 subvariant as compared with the prototype vaccine, NVX-CoV2373, when administered as a fourth dose. Safety data were consistent with the established safety profile of NVX-CoV2373.

Since the COVID-19 outbreak in late 2019, several variants of SARS-CoV-2 (eg, Alpha, Beta, Gamma, and Delta) have emerged with mutations in key antigenic sites in the receptor-binding domain and spike protein. In late 2021, the Omicron variant emerged as the dominant circulating SARS-CoV-2 virus globally, replacing earlier strains/variants. The emergence and propagation of SARS-CoV-2 variants, such as the Omicron sublineages, have complicated the COVID-19 vaccine landscape. Initial actions to stay ahead of SARS-CoV-2 evolution included directives from the US Food and Drug Administration (FDA) to develop vaccines containing an Omicron component [1, 2].

Large phase 3 clinical trials for prototype COVID-19 vaccines were conducted prior to the extensive prevalence of variant strains [3–6]. High vaccine efficacy against the ancestral (Wuhan) strain of SARS-CoV-2 was reported for BNT162b2 (Pfizer/BioNTech, July 2020–November 2020) [3], mRNA-1273 (Moderna, July 2020–October 2020) [4], the UK-based trial of the Matrix-M–adjuvanted recombinant spike (rS) protein COVID-19 vaccine NVX-CoV2373 [5], and the pivotal US trial of NVX-CoV2373 [6]. Multiple recent studies have demonstrated that newly emerging Omicron sublineages are less efficiently neutralized than the ancestral SARS-CoV-2 strain by approved prototype COVID-19 mRNA vaccines, including the BNT162b2 and mRNA-1273 vaccines [7–9]. In June 2022, regulatory bodies instructed COVID-19 vaccine manufacturers to develop bivalent vaccines consisting of the ancestral and Omicron BA.4/BA.5 subvariant strains to potentially provide increased protection from COVID-19 infection [1]; notably, a number of manufacturers already had bivalent vaccines consisting of the ancestral and Omicron BA.1 subvariant strains in development. However, sparse real-world data exist about the comparative effectiveness of monovalent vs bivalent SARS-CoV-2 vaccines. In June 2023 during a Vaccines and Related Biological Products Advisory Committee Meeting, the FDA recommended development of a monovalent Omicron XBB sublineage vaccine for 2023 to 2024 [10]. Currently, the US FDA has granted emergency use authorization of 2 mRNA-based and 1 recombinant protein subunit–based monovalent vaccines targeting the monovalent Omicron XBB sublineage [11, 12]. The World Health Organization’s Technical Advisory Group on COVID-19 Vaccine Composition, as well as the European Center for Disease Prevention and Control and the European Medicines Agency, has similar recommendations for updating vaccines to target XBB strains [13, 14].

As part of Omicron-targeted vaccine evaluation, Novavax produced a novel Omicron BA.1 subvariant–specific vaccine (NVX-CoV2515) based on the same rS protein technology as its authorized prototype vaccine, NVX-CoV2373. NVX-CoV2515 is also a coformulated product consisting of full-length prefusion recombinant S protein trimers with the saponin-based adjuvant Matrix-M. NVX-CoV2515 was prepared for use alone or in combination with the prototype vaccine (NVX-CoV2373) as a bivalent mixture to determine whether it would enhance or broaden antibody responses across variants of concern. To provide real-world applicable data, the population planned for investigation included participants who had already received 3 prior vaccinations with mRNA-based vaccines produced by Moderna and Pfizer BioNTech (mRNA-1273 and BNT162b2, respectively).

Here, we describe interim results from an ongoing clinical trial evaluating the immunogenicity and safety of an Omicron BA.1–containing monovalent vaccine (NVX-CoV2515) and a bivalent vaccine (NVX-CoV2373 + NVX-Cov2515) as compared with the original NVX-CoV2373 booster. The goal of this interim analysis was to determine if NVX-CoV2515 induces superior antibody responses to the Omicron BA.1 subvariant when compared with the antibody response induced by NVX-CoV2373.

## METHODS

As part of a phase 3 randomized observer-blinded study, participants who received a regimen of 3 prior doses of mRNA-1273 and/or BNT162b2 (likely monovalent, but this information was not captured in the study) were randomized 1:1:1 to receive NVX-CoV2373, NVX-CoV2515, or a bivalent mixture as a heterologous fourth dose. Eligible participants were ≥18 and ≤64 years of age and received their last dose of mRNA vaccine ≥90 days prior to their planned study vaccination.

Prior and concomitant medical history was collected during the screening period, including self-reporting of prior SARS-CoV-2 infection, per the study protocol. Baseline SARS-CoV-2 positivity was assessed by real-time reverse transcriptase polymerase chain reaction (rRT-PCR) of nasal swab specimens and antinucleocapsid (anti-N) serologic testing. All eligible participants had to be rRT-PCR negative for SARS-CoV-2; however, they could be included if they were anti-N positive (discussed later).

Participants received randomized investigational vaccines containing 5 µg of SARS-CoV-2 rS protein and 50 µg of Matrix-M adjuvant administered via a 0.5-mL intramuscular injection. The bivalent vaccine was prepared on-site as a 1:1 mixture of NVX-CoV2373 and NVX-CoV2515. Following vaccination, participants utilized an electronic diary to record daily solicited local reactions (tenderness, pain, redness, or swelling) and systemic reactions (fatigue, headache, muscle pain, malaise, joint pain, nausea/vomiting, or fever) for 7 days. Unsolicited adverse events (AEs) were collected for 28 days following vaccination: serious AEs (SAEs), AEs of special interest (including potentially immune-mediated medical conditions, myocarditis/pericarditis, and complications specific to COVID-19), and medically attended AEs. Data were analyzed from 3 participant analysis sets:

Safety analysis set: all participants who provided consent, were randomized, and received the study vaccine.
Per-protocol 1 analysis set (PP1): participants who received the study vaccine, had serology results for baseline and an analyzed time point, were negative at baseline for SARS-CoV-2 (determined by anti-N antibodies or rRT-PCR), and had no major protocol violations or events (eg, COVID-19 infection) that could affect immune responses.
Per-protocol 2 analysis set (PP2): the PP1 population but excluding the requirement for participants to have a negative baseline anti-N result (ie, required only rRT-PCR negativity).

PP1 and PP2 results were determined for each strain/subvariant, serology assay, and study visit.

Immune responses were assessed at 14 and 28 days following vaccination. Serum collected at each time point was analyzed with live virus neutralization [15], anti-spike immunoglobulin G (IgG) antibody [15], and pseudovirus neutralization assays [16]. MN titers were calculated by a visual cytopathic effect scoring method, as described previously [17]. Live virus neutralization assays provided microneutralization with an inhibitory concentration of 50% (MN_50_) data for the Omicron BA.1 sublineage (day 14, primary endpoint) and ancestral strain (validated by 360biolabs). The 95% CIs for geometric mean titer (GMT) and geometric mean fold rise were based on a t-distribution of the log-transformed values. The GMT ratio at day 14 and the 2-sided 95% CIs were computed with the analysis of covariance, with the vaccine group as the fixed effect and the titer at day 0 as the covariate under a 2-sided type I error rate of 0.05. Statistical significance was achieved if the lower bound of the 2-sided 95% CI was above unity (ie, >1). Seroresponse rates (SRRs) in MN_50_ titers (defined as a ≥4-fold increase from baseline values) of the Omicron BA.1 subvariant at day 14 following study vaccination were analyzed as part of the primary endpoint. SRRs were evaluated for ancestral strain. Two-sided exact binomial 95% CIs were calculated by the Clopper-Pearson method. The difference in SRR between groups (expressed as NVX-CoV2515 minus NVX-CoV2373) was calculated, with the 95% CI for the difference based on the method of Miettinen and Nurminen. For the analysis of difference of SRRs, based on an assumption of 80% SRR for NVX-CoV2373 and 85% SRR for NVX-CoV2515, there was 90% power to conclude noninferiority with a margin of −5% (NVX-CoV2515 relative to NVX-CoV2373). Participant serum antibody concentrations were measured via a previously validated assay [18].

For the GMT ratio analysis, a ratio of 1.5, an SD of 0.6 for log_10_-transformed neutralization titers based on data from previous studies, a 15% nonevaluable allowance, and an overall 1-sided type I error of 2.5% were assumed.

The trial protocol was approved by the Alfred Hospital Ethics Committee and the Bellberry Human Research Ethics Committee and is registered on ClinicalTrials.gov (NCT05372588). This study was performed in accordance with the International Conference on Harmonization’s good clinical practice guidelines. All participants provided informed consent prior to study participation.

Additional trial details are described in the supplementary methods.

## RESULTS

From 31 May 2022 to 17 July 2022, 835 participants (from 19 study sites across Australia) were screened and 831 were randomized to 1 of 3 treatment groups. Of the randomized participants, 829 received vaccine: 286, 274, and 269 received NVX-CoV2515, NVX-CoV2373, and bivalent vaccine (safety analysis set), respectively (Supplementary Figure 1).

Demographic and other baseline characteristics of the participants in the safety analysis set were similar across all vaccine groups (Table 1, supplementary results). The median age was 41.0 to 42.0 years, the majority of participants in each group were female, and most were White and of Australian ethnicity. Baseline SARS-CoV-2 exposure was substantial, with ≥50.9% of participants (NVX-CoV2515, 149/286; NVX-CoV2373, 145/274; bivalent, 137/269) testing positive by rRT-PCR or anti-N serology at the time of vaccination (day 0). These participants were excluded from the primary endpoint analysis of the PP1 population, but they were included in complementary analyses of the PP2 population to provide data more representative of a “real-world” population. Demographic and other baseline characteristics of the PP1 and PP2 populations were similar to those in the safety analysis set and were generally well balanced across the treatment groups (Supplementary Table 1).

**Table 1.** Demographics and Baseline Disease Characteristics: Safety Analysis Set

	Participants, No. (%)^a^		
Parameter	NVX-CoV2515 (n = 286)	NVX-CoV2373 (n = 274)	Bivalent^b^ (n = 269)
Age,^c^ y			
Mean (SD)	40.4 (12.1)	40.1 (11.5)	39.9 (12.4)
Median	42.0	41.0	41.0
Range	18–64	18–64	18–64
Sex			
Male	133 (46.5)	131 (47.8)	118 (43.9)
Female	153 (53.5)	143 (52.2)	151 (56.1)
Race			
White	233 (81.5)	215 (78.5)	220 (81.8)
Black or African American	0	2 (0.7)	0
Aboriginal Australian	2 (0.7)	1 (0.4)	2 (0.7)
Native Hawaiian or other Pacific Islander	1 (0.3)	0	1 (0.4)
Asian	37 (12.9)	45 (16.4)	39 (14.5)
Mixed origin	5 (1.7)	3 (1.1)	1 (0.4)
Other	8 (2.8)	8 (2.9)	6 (2.2)
Not reported	0	0	0
Ethnicity			
Australian	252 (88.1)	236 (86.1)	233 (86.6)
Aboriginal/Torres Strait Islanders	4 (1.4)	3 (1.1)	2 (0.7)
Hispanic or Latino	6 (2.1)	8 (2.9)	6 (2.2)
Not reported	12 (4.2)	15 (5.5)	17 (6.3)
Unknown	10 (3.5)	11 (4.0)	9 (3.3)
Missing	2 (0.7)	1 (0.4)	2 (0.7)
BMI,^d^ kg/m^2^			
No.	284	270	267
Mean (SD)	28.07 (6.4)	28.01 (5.3)	27.40 (5.7)
Median	26.9	27.5	26.3
Range	18.1–55.8	17.4–47.2	17.7–50.1
BMI category, kg/m^2^			
Underweight, <18.0	0	3 (1.1)	2 (0.7)
Normal, 18.0–24.9	106 (37.1)	75 (27.4)	104 (38.7)
Overweight, 25.0–29.9	87 (30.4)	108 (39.4)	90 (33.5)
Obese, ≥30.0	91 (31.8)	84 (30.7)	71 (26.4)
Missing	2 (0.7)	4 (1.5)	2 (0.7)
Regimen of previous COVID-19 vaccine			
Moderna	0	2 (0.7)	5 (1.9)
Pfizer-BioNTech	213 (74.5)	214 (78.1)	200 (74.3)
Mixed	73 (25.5)	58 (21.2)	64 (23.8)
Moderna-Moderna-Pfizer	1 (0.3)	1 (0.4)	0
Moderna-Pfizer-Pfizer	2 (0.7)	0	1 (0.4)
Moderna-Pfizer-Moderna	0	0	0
Pfizer-Pfizer-Moderna	70 (24.5)	56 (20.4)	63 (23.4)
Pfizer-Moderna-Moderna	0	1 (0.4)	0
Pfizer-Moderna-Pfizer	0	0	0
Previous COVID-19			
Yes	18 (6.3)	19 (6.9)	17 (6.3)
No	268 (93.7)	255 (93.1)	252 (93.7)
Qualitative anti-N			
Positive	145 (50.7)	141 (51.5)	134 (49.8)
Negative	141 (49.3)	133 (48.5)	135 (50.2)
rRT-PCR			
Positive	11 (3.8)	12 (4.4)	14 (5.2)
Negative	275 (96.2)	262 (95.6)	255 (94.8)
Anti-N/rRT-PCR^e^			
Positive	149 (52.1)	145 (52.9)	137 (50.9)
Negative	137 (47.9)	129 (47.1)	132 (49.1)
Time between last COVID-19 vaccine and booster dose of study vaccine, d			
Mean (SD)	178.2 (38.5)	182.4 (36.4)	178.7 (36.6)
Median	177.0	182.0	180.0
Range	84–440	91–329	77–313
Interval between last COVID-19 vaccine and booster dose of study vaccine, d			
<90	1 (0.3)	0	1 (0.4)
90–120	15 (5.2)	15 (5.5)	18 (6.7)
>120–150	43 (15.0)	35 (12.8)	36 (13.4)
>150–180	98 (34.3)	81 (29.6)	81 (30.1)
>180–210	87 (30.4)	97 (35.4)	94 (34.9)
>210–240	26 (9.1)	32 (11.7)	25 (9.3)
>240–270	10 (3.5)	9 (3.3)	11 (4.1)
>270–300	4 (1.4)	2 (0.7)	1 (0.4)
>300–330	1 (0.3)	3 (1.1)	2 (0.7)
>330–360	0	0	0
>360	1 (0.3)	0	0

Each participant received 5 µg of SARS-CoV-2 recombinant spike protein nanoparticle vaccine with 50 µg of Matrix-M adjuvant. Participants in the safety analysis set are counted according to the treatment received to accommodate for treatment errors. The sample number for continuous parameters represents the number of participants with nonmissing values.

Abbreviations: anti-N, antinucleocapsid; BMI, body mass index; rRT-PCR, real-time reverse transcriptase polymerase chain reaction.

^a^Data are presented as No. (%) unless noted otherwise.

^b^NVX CoV2373 + NVX-CoV2515.

^c^Age was calculated at the time of informed consent.

^d^BMI was calculated as weight (kilograms) divided by height squared (meters). Percentages were based on the safety analysis set within each treatment and overall.

^e^Participants with either anti-N or rRT-PCR are reported.

### PP1 Analysis Set

**Figure 1.**
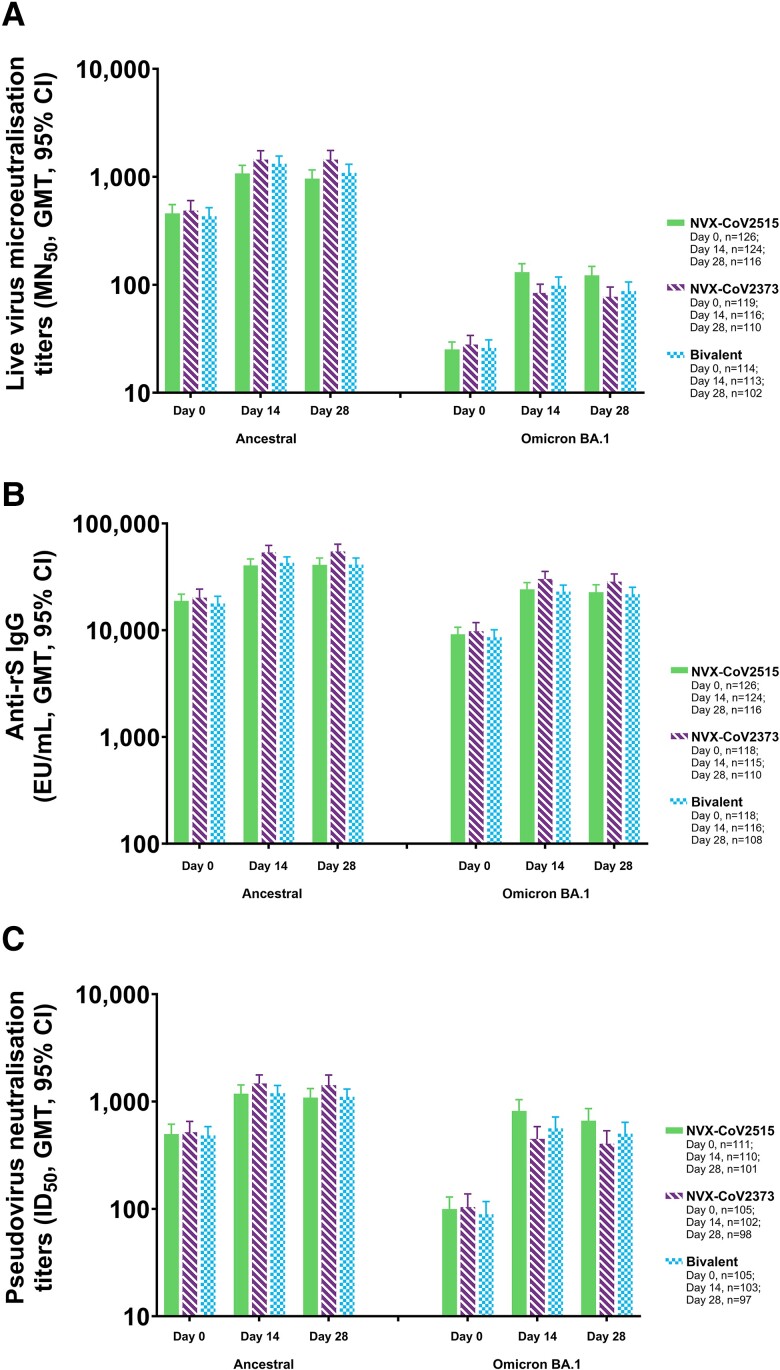
Immunogenicity against ancestral and BA.1 variant strains of SARS-CoV-2 after booster vaccination with NVX-CoV2515, NVX-CoV2373, or bivalent NVX-CoV2373 + NVX-CoV2515: Per-protocol 1 analysis set. *A–C*, Microneutralization titers, anti-spike IgG concentrations, and pseudovirus neutralization titers for the ancestral and BA.1 variant. EU, enzyme-linked immunosorbent assay unit; GMT, geometric mean titer; ID_50_, inhibitory dilution with a 50% concentration; IgG, immunoglobulin G; MN_50_, microneutralization with an inhibitory concentration of 50%; rS, recombinant spike.

Among participants with no evidence of SARS-CoV-2 exposure at baseline (PP1 population), strong immune responses were observed following administration with all 3 investigational vaccines at 14 days postvaccination (Table 2, Supplementary Table 2). At day 14, microneutralization assay GMTs against the Omicron BA.1 sublineage were the highest in participants receiving NVX-CoV2515, followed by those receiving the bivalent vaccine and NVX-CoV2373 (MN_50_ titers [95% CI]: 130.8 [109.2–156.7], 97.9 [81.3–117.9], and 83.9 [69.6–101.2], respectively; Figure 1). Formal comparison of NVX-CoV2515 and NVX-CoV2373 resulted in a GMT ratio of 1.6 (95% CI, 1.33–2.03), indicating a significant difference between the vaccines. NVX-CoV2515 induced a noninferior SRR against the Omicron BA.1 subvariant virus vs NVX-CoV2373 (73.4% [91/124] vs 50.9% [59/116]) at day 14, with a difference in SRRs of 22.5% (95% CI, 10.3–34.2). MN_50_ GMTs for the ancestral strain microneutralization assay were somewhat lower in participants receiving NVX-CoV2515 as compared with the bivalent vaccine and NVX-CoV2373 alone (MN_50_ titers [95% CI]: 1076.3 [908.4–1275.4], 1319.9 [1120.1–1555.3], and 1442.5 [1192.4–1745.0], respectively). Similar findings were seen at day 28.

**Table 2.** Key Assay Endpoints for Serum Microneutralization Titers and Pseudovirus Neutralization Titers Against the Ancestral and Omicron BA.1 Variant Strain After a Heterologous Fourth Booster Dose: Per-Protocol 1 Analysis Set

	Vaccine Group^a^					
	NVX-CoV2515 (n = 126)		NVX-CoV2373 (n = 119)		Bivalent (n = 118)	
	Ancestral	BA.1	Ancestral	BA.1	Ancestral	BA.1
Microneutralization titers						
Day 0: baseline^b^						
No.	126	126	119	119	114	114
GMT (95% CI),^c^ MN_50_	457.6 (378.2–553.5)	25.2 (21.5–29.5)	486.7 (393.3–602.3)	27.9 (22.9–33.9)	431.1 (359.4–517.0)	26.0 (21.8–30.9)
Day 14						
No.	124	124	116	116	113	113
GMT (95% CI), MN_50_	1076.3 (908.4–1275.4)	130.8 (109.2–156.7)	1442.5 (1192.4–1745.0)	83.9 (69.6–101.2)	1319.9 (1120.1–1555.3)	97.9 (81.3–117.9)
SRR ≥4-fold increase, No. (%)^d^	54 (43.5)	91 (73.4)	57 (49.1)	59 (50.9)	64 (56.6)	74 (65.5)
95% CI^e^	34.7–52.7	64.7–80.9	39.7–58.6	41.4–60.3	47.0–65.9	56.0–74.2
Day 28						
No.	116	116	110	110	102	102
GMT (95% CI), MN_50_	960.8 (798.0–1156.9)	122.3 (101.0–148.0)	1442.8 (1188.1–1752.1)	77.5 (63.1–95.3)	1087.4 (906.5–1304.3)	87.4 (72.0–106.1)
SRR ≥4-fold increase, No. (%)	37 (31.9)	86 (74.1)	54 (49.1)	52 (47.3)	42 (41.2)	56 (54.9)
95% CI	23.6–41.2	65.2–81.8	39.4–58.8	37.7–57.0	31.5–51.4	44.7–64.8
Pseudovirus neutralization titers, ID_50_						
Day 0: baseline						
No.	111	111	105	105	105	105
GMT (95% CI), ID_50_	496.7 (401.0–615.2)	99.6 (77.1–128.8)	517.1 (409.3–653.1)	103.6 (77.9–137.8)	483.3 (400.7–583.0)	89.2 (68.2–116.9)
Day 14						
No.	110	110	102	102	103	103
GMT (95% CI), ID_50_	1181.1 (976.6–1428.3)	816.1 (639.9–1040.7)	1473.0 (1227.1–1768.3)	449.1 (345.3–584.2)	1195.1 (1012.1–1411.2)	562.0 (439.6–718.6)
SRR ≥4-fold increase, No. (%)	21 (19.1)	81 (73.6)	25 (24.5)	48 (47.1)	25 (24.3)	68 (66.0)
95% CI	12.2–27.7	64.4–81.6	16.5–34.0	37.1–57.2	16.4–33.7	56.0–75.1
Day 28						
No.	101	101	98	98	97	97
GMT (95% CI), ID_50_	1085.8 (891.5–1322.4)	661.6 (509.7–858.7)	1420.2 (1141.9–1766.2)	404.1 (305.4–534.7)	1106.1 (937.7–1304.8)	502.1 (393.7–640.4)
SRR ≥4-fold increase, No. (%)	20 (19.8)	69 (68.3)	29 (29.6)	46 (46.9)	18 (18.6)	58 (59.8)
95% CI	12.5–28.9	58.3–77.2	20.8–39.7	36.8–57.3	11.4–27.7	49.3–69.6

Values less than the LLOQ were replaced by 0.5 × LLOQ.

Abbreviations: GMT, geometric mean titer; ID_50_, inhibitory dilution with a 50% concentration; LLOQ, lower limit of quantitation; MN_50_, microneutralization with an inhibitory concentration of 50%; SRR, seroresponse rate.

^a^NVX-CoV2515, 5-μg Omicron BA.1 recombinant spike. NVX-CoV2373, 5-μg ancestral recombinant spike. Bivalent, 2.5-μg ancestral recombinant spike + 2.5-μg Omicron BA.1 recombinant spike.

^b^Baseline was defined as the last nonmissing assessment prior to first vaccination.

^c^The 95% CI for GMT was calculated by the t-distribution of the log-transformed values and then back-transformed to the original scale for presentation.

^d^SRR was defined as the percentage of participants at each postvaccination visit with a titer having a ≥4-fold rise in MN_50_ or ID_50_ level. Percentage is based on the number of participants within each visit with nonmissing data.

^e^The 95% CI for SRR was calculated per the exact Clopper-Pearson method.

Anti-spike IgG assay data indicate that the highest concentrations of Omicron BA.1 IgG are achieved with NVX-CoV2373, highlighting the vaccine's cross-reactive nature with the Omicron BA.1 sublineage. Anti-spike IgG antibody levels (geometric mean enzyme-linked immunosorbent assay units [EUs; 95% CI]) for the BA.1 assay were 30 170.9 (25 663.7–35 469.6), 24 174.8 (20 943.6–27 904.6), and 23 045.5 (20 113.5–26 404.8) EU/mL for NVX-CoV2373, NVX-CoV2515, and the bivalent vaccine, respectively (Figure 1). A somewhat similar pattern of response was seen with the ancestral strain anti-spike IgG assay (supplementary results).

Pseudovirus neutralization GMTs against the Omicron BA.1 sublineage were the highest in participants receiving NVX-CoV2515, followed by those receiving the bivalent vaccine and NVX-CoV2373 (ID_50_ titers [inhibitory dilution with a 50% concentration; 95% CI]: 816.1 [639.9–1040.7], 562.0 [439.6–718.6], and 449.1 [345.3–584.2], respectively; Figure 1). ID_50_ GMTs for the ancestral strain pseudovirus neutralization assay were somewhat lower in participants receiving NVX-CoV2515 and the bivalent vaccine as compared with NVX-CoV2373 (Table 2, Supplementary Table 2). For all 3 assays against the ancestral strain, SRRs were lower for NVX-CoV2515 than for NVX-CoV2373.

### PP2 Analysis Set

**Figure 2.**
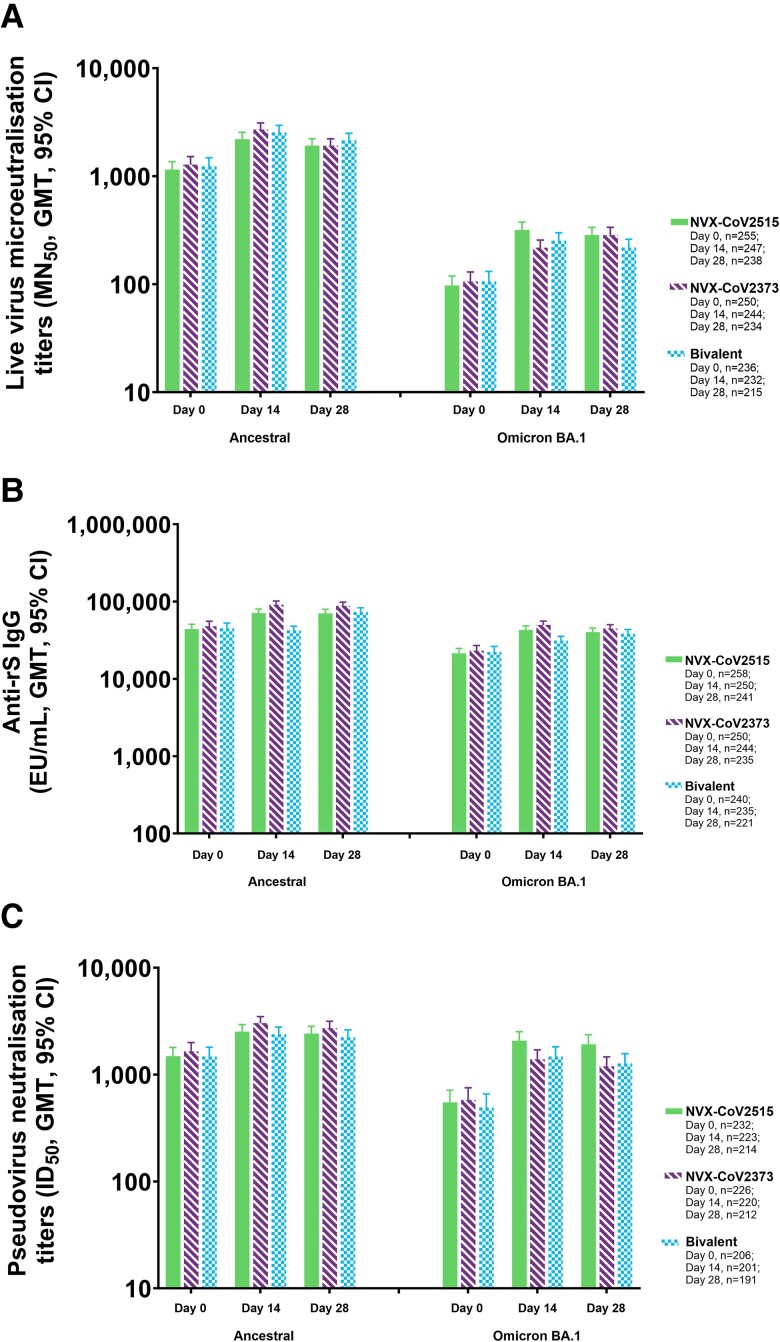
Immunogenicity against ancestral and BA.1 variant strains of SARS-CoV-2 after booster vaccination with NVX-CoV2515, NVX-CoV2373, or bivalent NVX-CoV2373 + NVX-CoV2515: Per-protocol 2 analysis set. *A–C*, Microneutralization titers, anti-spike IgG concentrations, and pseudovirus neutralization titers for the ancestral and BA.1 variant. EU, enzyme-linked immunosorbent assay unit; GMT, geometric mean titer; ID_50_, inhibitory dilution with a 50% concentration; IgG, immunoglobulin G; MN_50_, microneutralization with an inhibitory concentration of 50%; rS, recombinant spike.

Microneutralization assay GMTs against the Omicron BA.1 sublineage for participants in the PP2 population, which included those with a positive baseline SARS-CoV-2 result, were the highest in participants receiving NVX-CoV2515, followed by those receiving the bivalent vaccine and NVX-CoV2373 (MN_50_ titers [95% CI]: 318.2 [269.8–375.3], 252.7 [213.1–299.7], and 218.1 [186.0–255.7], respectively; Figure 2). NVX-CoV2515 induced a noninferior SRR against the Omicron BA.1 subvariant virus vs NVX-CoV2373 (54.3% [134/247] vs 32.0% [78/244]) at day 14, with similar results on day 28 (Table 3, Supplementary Table 3). MN_50_ GMTs for the ancestral strain microneutralization assay were somewhat lower in participants receiving NVX-CoV2515 as compared with the bivalent and NVX-CoV2373 vaccines (MN_50_ titers [95% CI]: 2206.2 [1910.0–2548.4], 2544.7 [2194.5–2950.9], and 2702.0 [2347.9–3109.4], respectively).

**Table 3.** Key Assay Endpoints for Serum Microneutralization Titers and Pseudovirus Neutralization Titers Against the Ancestral and Omicron BA.1 Variant Strain After a Heterologous Fourth Booster Dose: Per-Protocol 2 Analysis Set

	Vaccine Group^a^					
	NVX-CoV2515 (n = 286)		NVX-CoV2373 (n = 274)		Bivalent (n = 269)	
	Ancestral	BA.1	Ancestral	BA.1	Ancestral	BA.1
Microneutralization titers						
Day 0: baseline^b^						
No.	255	255	250	250	236	236
GMT (95% CI),^c^ MN_50_	1151.3 (973.6–1361.4)	97.3 (79.6–118.9)	1280.0 (1078.1–1519.7)	105.9 (86.4–129.7)	1232.0 (1026.0–1479.5)	106.1 (85.8–131.1)
Day 14						
No.	247	247	244	244	232	232
GMT (95% CI), MN_50_	2206.2 (1910.0–2548.4)	318.2 (269.8–375.3)	2702.0 (2347.9–3109.4)	218.1 (186.0–255.7)	2544.7 (2194.5–2950.9)	252.7 (213.1–299.7)
SRR ≥4-fold increase, No. (%)^d^	79 (32.0)	134 (54.3)	80 (32.8)	78 (32.0)	83 (35.8)	95 (40.9)
95% CI^e^	26.2–38.2	47.8–60.6	26.9–39.1	26.2–38.2	29.6–42.3	34.6–47.6
Day 28						
No.	238	238	234	234	215	215
GMT (95% CI), MN_50_	1918.8 (1657.9–2220.6)	284.8 (241.8–335.4)	2456.0 (2145.2–2811.8)	195.7 (165.7–231.2)	2144.0 (1842.3–2495.2)	218.7 (183.1–261.3)
SRR ≥4-fold increase, No. (%)	56 (23.5)	125 (52.5)	68 (29.1)	65 (27.8)	59 (27.4)	72 (33.5)
95% CI	18.3–29.4	46.0–59.0	23.3–35.3	22.1–34.0	21.6–33.9	27.2–40.2
Pseudovirus neutralization titers, ID_50_						
Day 0: baseline						
No.	232	232	226	226	206	206
GMT (95% CI), ID_50_	1491.2 (1237.3–1797.1)	548.4 (421.7–713.2)	1646.5 (1357.6–1996.8)	579.2 (445.6–752.8)	1476.4 (1208.2–1804.1)	491.9 (366.8–659.6)
Day 14						
No.	223	223	220	220	201	201
GMT (95% CI), ID_50_	2514.9 (2149.8–2942.0)	2071.5 (1701.1–2522.5)	3027.9 (2625.7–3491.6)	1390.3 (1134.6–1703.8)	2390.1 (2050.2–2786.3)	1480.4 (1202.8–1822.1)
SRR ≥4-fold increase, No. (%)	25 (11.2)	102 (45.7)	26 (11.8)	53 (24.1)	25 (12.4)	71 (35.3)
95% CI	7.4–16.1	39.1–52.5	7.9–16.8	18.6–30.3	8.2–17.8	28.7–42.4
Day 28						
No.	214	214	212	212	191	191
GMT (95% CI), ID_50_	2411.6 (2055.6–2829.2)	1918.0 (1558.2–2361.0)	2716.9 (2340.0–3154.5)	1195.7 (974.9–1466.6)	2236.0 (1909.8–2617.9)	1272.4 (1033.5–1566.6)
SRR ≥4-fold increase, No. (%)	22 (10.3)	84 (39.3)	29 (13.7)	48 (22.6)	19 (9.9)	61 (31.9)
95% CI	6.6–15.2	32.7–46.1	9.4–19.1	17.2–28.9	6.1–15.1	25.4–39.1

Values less than LLOQ were replaced by 0.5 × LLOQ.

Abbreviations: GMT, geometric mean titer; ID_50_, inhibitory dilution with a 50% concentration; LLOQ, lower limit of quantitation; SRR, seroresponse rate.

^a^NVX-CoV2515, 5-μg Omicron BA.1 recombinant spike. NVX-CoV2373, 5-μg ancestral recombinant spike. Bivalent, 2.5-μg ancestral recombinant spike + 2.5-μg Omicron BA.1 recombinant spike.

^b^Baseline was defined as the last nonmissing assessment prior to first vaccination.

^c^The 95% CI for GMT was calculated by the t-distribution of the log-transformed values and then back-transformed to the original scale for presentation.

^d^SRR was defined as the percentage of participants at each postvaccination visit with a titer having a ≥4-fold rise in MN_50_ or ID_50_ level. Percentage is based on the number of participants within each visit with nonmissing data.

^e^The 95% CI for SRR was calculated per the exact Clopper-Pearson method.

As in the PP1 population, anti-spike IgG assay data indicate the highest levels of BA.1 IgG are achieved with vaccination through NVX-CoV2373, indicating cross-reactivity with the Omicron BA.1 sublineage (Supplementary Table 3). Anti-spike IgG EUs (95% CI) for the BA.1 assay were 49 727.7 (44 331.1–55 781.1), 42 835.5 (37 883.8–48 434.4), and 42 462.1 (37 628.9–47 916.2) EU/mL for NVX-CoV2373, NVX-CoV2515, and the bivalent vaccine, respectively; a relatively similar pattern of response was seen with the ancestral strain anti-spike IgG assay (Figure 2, supplementary results).

For all 3 assays against the ancestral strain, SRRs were lower for NVX-CoV2515 than for NVX-CoV2373 (Table 3, Supplementary Table 3).

### Safety Analysis Set

**Figure 3.**
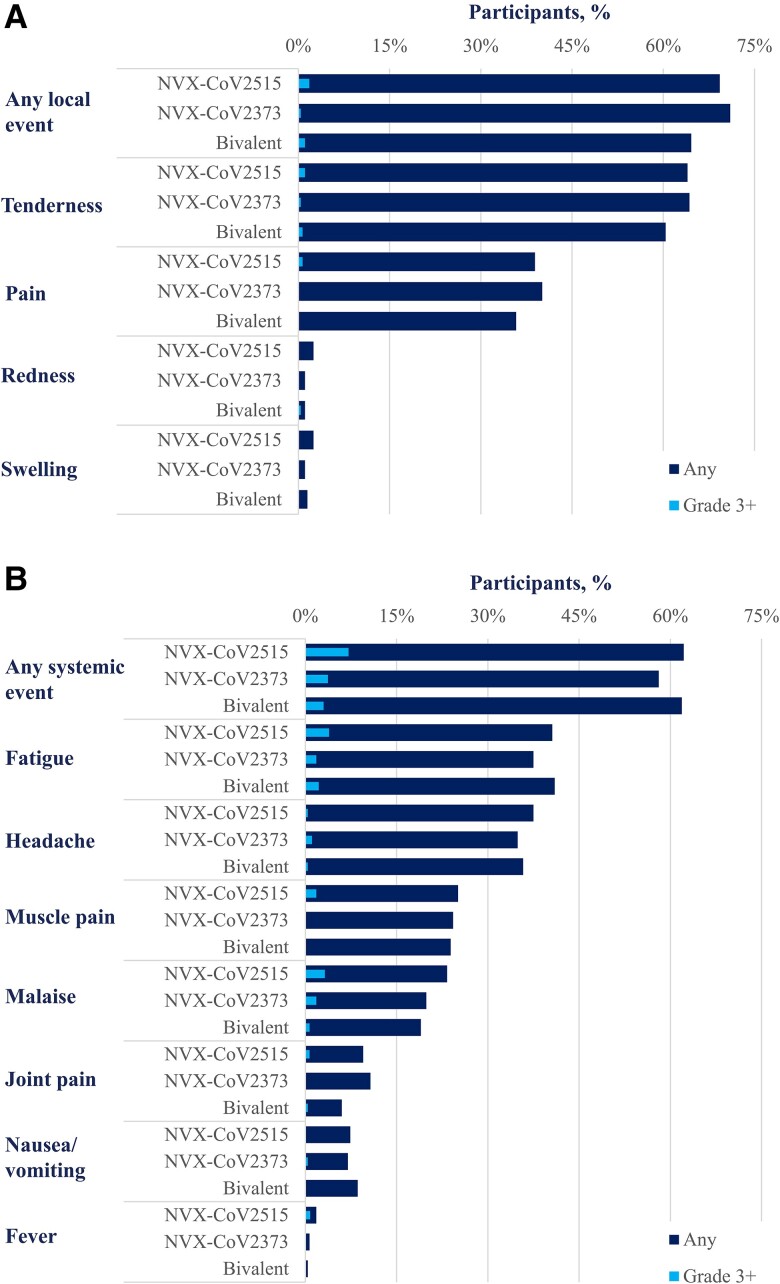
Solicited adverse events within 7 days of vaccination: safety analysis set. Frequencies of solicited treatment-emergent adverse events: *A*, local; *B*, systemic.

The overall rates of solicited local and systemic reactions reported within 7 days after booster vaccination were similar across all 3 investigational products (Figure 3). For NVX-CoV2515, NVX-CoV2373, and the bivalent vaccine, rates of solicited local events of any grade were 69.3% (196/286; grade ≥3, 1.8% [5/286]), 71.0% (193/274; 0.4% [1/274]), and 64.6% (173/269; 1.1% [3/269]), respectively. No grade 4 solicited local treatment-emergent AEs (TEAEs) were reported. Local reactions were generally short-lived, with a median duration of 1.0 day for all events except tenderness (2.0 days). Solicited systemic reactions were similar across the groups with event rates (grade ≥3 rates) of 62.2% (7.3%), 58.1% (3.7%), and 61.9% (3.0%) for NVX-CoV2515, NVX-CoV2373, and the bivalent vaccine, respectively. There was 1 grade 4 solicited systemic TEAE (fever) in the NVX-CoV2515 group. Solicited systemic reactions were transient, with a median duration of 1.0 day for all events except fatigue, which had a median duration of 2.0 days.

Through 28 days after vaccination, unsolicited TEAEs occurred in 34.3% (98/286), 38.0% (104/274), and 33.8% (91/269) of participants in the NVX-CoV2515, NVX-CoV2373, and bivalent vaccine groups, respectively (Table 4). Unsolicited serious TEAEs occurred in 0.3% (1/286) and 0.4% (1/274) of participants in the NVX-CoV2515 and NVX-CoV2373 groups, though neither event was reported as vaccine related. No unsolicited serious TEAEs occurred in the bivalent vaccine group. There were no treatment-related medically attended AEs or potential immune-mediated medical conditions in any vaccine group.

**Table 4.** Overall Unsolicited Treatment-Emergent Adverse Events Through 28 Days After Vaccination: Safety Analysis Set

	Participants, No. (%)		
Parameter	NVX-CoV2515 (n = 286)	NVX-CoV2373 (n = 274)	Bivalent (n = 269)
Solicited TEAEs			
Local^a^	196 (69.3)	193 (71.0)	173 (64.6)
Grade ≥3	5 (1.8)	1 (0.4)	3 (1.1)
Systemic^b^	176 (62.2)	158 (58.1)	166 (61.9)
Grade ≥3	21 (7.4)	10 (3.7)	8 (3.0)
Unsolicited TEAEs			
Any	98 (34.3)	104 (38.0)	91 (33.8)
Treatment related	14 (4.9)	8 (2.9)	9 (3.3)
Severe	0	4 (1.5)	0
Treatment related severe	0	0	0
Serious	1 (0.3)	1 (0.4)	0
Treatment related	0	0	0
Any unsolicited TEAE leading to			
Vaccination discontinuation	1 (0.3)	1 (0.4)	0
Treatment related	1 (0.3)	0	0
Study discontinuation	0	1 (0.4)	0
Treatment related	0	0	0
Any unsolicited treatment-emergent MAAE	14 (4.9)	18 (6.6)	13 (4.8)
Treatment related	1 (0.3)	0	0
Treatment related serious	0	0	0
Severe	0	3 (1.1)	0
Treatment related severe	0	0	0
Any unsolicited AESI			
PIMMC	0	1 (0.4)	0
Treatment related	0	0	0
Complications due to COVID-19	0	0	0
Any myocarditis/pericarditis	0	0	0

Participants in the safety analysis set are counted according to the treatment received to accommodate for treatment errors.

Abbreviations: AESI, adverse event of special interest; MAAE, medically attended adverse event; PIMMC, potentially immune-mediated medical condition; TEAE, treatment-emergent adverse event.

^a^Solicited local events included tenderness, pain, redness, and swelling.

^b^Solicited systemic events included fatigue, headache, muscle pain, malaise, joint pain, nausea/vomiting, and fever.

## DISCUSSION

In this report, we describe the first immunogenicity and safety data for Omicron-specific (NVX-CoV2515) and bivalent SARS-CoV-2 rS protein subunit vaccines. These results are from an interim analysis of an ongoing phase 3 randomized observer-blinded clinical trial in participants who previously received a regimen of 3 doses of prototype mRNA vaccine.

The primary endpoint analysis demonstrated that the Omicron BA.1–specific vaccine, NVX-CoV2515, produced a superior neutralizing antibody response (MN_50_) against the Omicron BA.1 subvariant when compared with the prototype vaccine, NVX-CoV2373, and met the noninferiority criterion for SRR vs NVX-CoV2373 at day 14 following booster administration, thereby successfully achieving the study's primary objective.

While NVX-CoV2515 demonstrated superior MN_50_ and ID_50_ titers against the matched Omicron BA.1 strain than NVX-CoV2373, this finding was not seen for IgG titers. Similarly, NVX-CoV2373 elicited higher MN_50_ and pseudovirus ID_50_ responses against the matched ancestral strain than the Omicron-based vaccines. Trends with the MN_50_ assays were maintained in participants with no evidence of previous infection (PP1) and those with evidence of previous infection as determined by anti-N status at baseline (included in PP2). With regard to prevention of severe disease and hospitalization due to COVID-19, neutralizing and nonneutralizing antibodies play a role in vaccine efficacy [19]. Thus, lower titers for neutralizing antibodies do not necessarily indicate lower vaccine efficacy. Overall, neutralization titers (but not IgG titers) against Omicron BA.1 were higher with the bivalent vaccine and NVX-CoV2515 as compared with the prototype vaccine.

In a recent study, the immunogenicity of the mRNA-1273.214 bivalent vaccine (ancestral SARS-CoV-2 strain + Omicron BA.1) was investigated in individuals who previously received 3 doses of the prototype mRNA-1273 vaccine [20]. When compared with individuals who received the prototype mRNA.1273 vaccine as a fourth booster dose, those who received mRNA.1273.214 as a fourth dose exhibited higher binding antibody responses against Omicron BA.1 and Omicron BA.4/5 variants, resulting in the acknowledged superiority of mRNA.1273.214 to the mRNA-1273 prototype vaccine. An additional study conducted with the same bivalent mRNA vaccine showed that the Omicron BA.1–monovalent mRNA-1273.529 and bivalent mRNA-1273.214 vaccines elicited superior neutralizing antibody responses against Omicron BA.1 as compared with the prototype mRNA-1273 vaccine [21].

After receipt of a single dose of NVX-CoV2515, NVX-CoV2373, or bivalent vaccine, participant sera from the 3 study groups achieved anti-spike IgG antibody concentrations against the ancestral SARS-CoV-2 strain that were previously associated with vaccine efficacy levels of 88% to 95% in pivotal phase 3 studies of the prototype vaccine [6, 22]. However, as the correlates of protection were established in a study in which the ancestral Wuhan and alpha strains predominantly circulated, they may not be directly applicable to more recent Omicron subvariants. Anti-spike IgG antibody responses from the Omicron BA.1–specific assay were balanced across the 3 study groups regardless of baseline status of confirmed prior infection, thereby displaying similar benefits of NVX-CoV2515, the bivalent vaccine, and the prototype vaccine. The consistent anti-spike IgG responses (agnostic of strain) suggest the development of broadly cross-reacting IgG antibodies following administration of SARS-CoV-2 rS protein subunit vaccines, as the prototype, BA.1 variant, or bivalent vaccine.

Overall, the variant-specific vaccine (NVX-CoV2515) induced a superior neutralizing antibody response against the Omicron BA.1 subvariant when compared with the prototype vaccine, NVX-CoV2373. The NVX-CoV2515 and bivalent SARS-CoV-2 rS protein subunit vaccines demonstrated similar immunogenicity 14 days postvaccination, with no added benefits of using the bivalent of Omicron-adapted vaccines when compared with the prototype product (NVX-CoV2373) across several ancestral strain- and Omicron BA.1–specific immunoassays. Immunogenicity results at day 28 were generally similar to those at day 14.

The PP2 population assessed in this study most accurately represents a real-world population in which previous SARS-CoV-2 infection is not uncommon. In Australia as of November 2022, at least 66% of the population was estimated to have been previously infected by SARS-CoV-2 [23]. Anti-spike IgG antibody responses against the Omicron BA.1 subvariant for the PP2 population were generally 1.5- to 2-fold greater than those for the PP1 population. These results align with studies that assessed immune responses in participants with and without previous SARS-CoV-2 infection, which showed that neutralization of subvariant SARS-CoV-2 strains was higher after a booster with bivalent mRNA vaccine than after a booster dose with prototype mRNA vaccine [20, 24].

The incidence of solicited local and systemic reactogenicity reported in this study was consistent with previous studies of NVX-CoV2373, with pain/tenderness being the most common local solicited AE and fatigue the most common solicited systemic AE [6, 25]. Incidence rates for all local and systemic events were similar across all vaccine groups.

Incidences of unsolicited TEAEs and serious AEs were also unremarkable with respect to prior research on the prototype vaccine, and there were no reports of related medically attended AEs, potentially immune-mediated medical conditions, or SAEs. Collectively, these data were consistent with the safety profile of other variant-specific SARS-CoV-2 rS protein subunit vaccines or bivalent combination or with use as a heterologous booster in combination with mRNA vaccines.

Our study was subject to certain limitations. As these results are from an ongoing phase 3 study conducted with a limited sample size, the clinical efficacy of the booster dose was not evaluated. Safety follow-up in this study, at present, is limited to 28 days. As anti-N antibodies may wane over time [26, 27], some participants with undetected hybrid immunity may have been included in the PP1 study population. However, rRT-PCR testing at baseline and self-reporting of prior infection were used to address this limitation. Furthermore, it remains to be seen if the conclusions based on the prototype vs variant-specific vaccines in this study can be extrapolated to newer strains or vaccines. A recent study compared the neutralization activity of a bivalent BA.4/5 BNT162b2 vaccine with the prototype BNT162b2 vaccine against newly emerged Omicron sublineages descended from BA.2 and BA.4/BA.5 in persons who previously received 3 doses of BNT162b2. Data revealed that the bivalent BA.4/5 vaccine was more immunogenic than the original BNT162b2 monovalent vaccine against circulating Omicron sublineages [24]. Additionally, newer Omicron subvariants, such as BQ and XBB, showed marked evasion of vaccine-induced neutralization and evasion from monoclonal antibodies with known neutralization capability against the original Omicron variant [28]. Therefore, responses to the Omicron BA.5 variant after immunization with NVX-CoV2515, NVX-CoV2373, or bivalent vaccine and the effect of a subsequent booster dose at 3 months will be addressed in future work, given that BA.5 is more closely related phylogenetically to XBB and BQ than to the ancestral strain.

In conclusion, the variant-specific vaccine NVX-CoV2515 demonstrated superior neutralizing response against the matched Omicron BA.1 subvariant virus. The prototype and bivalent vaccines also induced robust immune responses to ancestral and Omicron subvariant strains of SARS-CoV-2 when administered as a fourth dose. Moreover, the safety profile of updated variant-specific SARS-CoV-2 rS protein subunit vaccines remained consistent with the prototype vaccine when administered as a heterologous booster dose following 3 vaccinations with mRNA vaccines.

## Supplementary Data

Supplementary materials are available at *The Journal of Infectious Diseases* online (http://jid.oxfordjournals.org/). Supplementary materials consist of data provided by the author that are published to benefit the reader. The posted materials are not copyedited. The contents of all supplementary data are the sole responsibility of the authors. Questions or messages regarding errors should be addressed to the author.

## Supplementary Material

jiad508_Supplementary_Data

## Notes

***Acknowledgments.*** We thank all the study participants who volunteered for this study. Editorial support was provided by Kelly Cameron, PhD, and Amanda Cox, PhD, of Ashfield MedComms, an Inizio company, supported by Novavax Inc.

***Author contributions.*** R. M. M., J. S. P., and N. P. were involved in the study design, data collection, and interpretation. S. G. performed the statistical analyses. All authors reviewed, commented on, and approved this manuscript prior to submission for publication. The authors were not precluded from accessing data in the study, and they accept responsibility to submit the manuscript for publication.

***Data sharing.*** Study information is available at https://clinicaltrials.gov/ct2/show/NCT05372588, and requests will be considered.

***Financial support.*** This work was supported by Novavax, Inc.
