# Supplementary material for: Immunogenicity and Safety of Heterologous Omicron BA.1 and Bivalent SARS-CoV-2 Recombinant Spike Protein Booster Vaccines: A Phase 3 Randomized Clinical Trial
Source: J Infect Dis. 2023 Nov 16;230(1):e4–e16. doi: 10.1093/infdis/jiad508 (PMC11272042; doi:10.1093/infdis/jiad508)
Supplement: jiad508_Supplementary_Data [file jiad508_supplementary_data.docx]

**Supplementary Methods**

rRT-PCR was determined by the Abbott RealTime Quantitative SARS-CoV-2 assay on the Abbott m2000 System. The Abbott RealTime SARS-CoV-2 assay is a dual target assay for the RdRp and N genes and is validated to quantitate SARS-CoV-2 nucleic acid in nasopharyngeal, oropharyngeal, and nasal swab samples. Anti-N analysis of the serum samples was performed using the Roche Elecsys® Anti-SARS-CoV-2 qualitative Emergency Use Authorization (EUA) assay using the Cobas e411 instrument at the University of Washington Retrovirology Laboratory, which is validated to qualitatively measure any detected antibodies to SARS-CoV-2 nucleocapsid protein in human serum and plasma. Diagnostic sensitivity and specificity of Anti-N assay were evaluated by testing twenty SARS-CoV-2 antibody positive clinical samples and twenty SARS-CoV-2 antibody negative clinical samples during assay validation. Due to inherent differences between technologies, 100% agreement is not expected. Only one (of 20, 5%) previously nonreactive sample was reactive on this assay and one (of 20, 5%) previously reactive sample was nonreactive on this assay. These results met the pre-established assay acceptability criteria of 95% of the observed results corresponding to the previous test results and show the specificity of the assay. Also, 792 potentially cross-reactive serum samples collected from persons with acute Epstein-Barr virus (105 samples), syphilis (62 samples), cytomegalovirus (85 samples) hepatitis C virus (60 samples) and many other infectious diseases including common cold panel, varicella zoster virus, influenza, etc, were also tested in the anti-N assay. Out of 792 potentially cross-reacting samples, 4 samples showed reactivity in the Elecsys Anti-SARS-CoV-2 assay resulting in an overall specificity of 99.5 %. All these results show the specificity of the anti-N assay used in our study.

The residual non-neutralized virus in the MN assay was detected via cytopathic effect assessed by microscopic scoring by a fully trained experienced subject matter expert. Two replicate wells per dilution were scored as either positive (SARS-CoV-2 cytopathology is present) or negative (healthy Vero E6 monolayer). The neutralization titer was expressed as the reciprocal of the highest dilution at which ≥50% of the replicate wells were protected from infection (MN50).

Serum anti-spike IgG enzyme linked immunosorbent assays (ELISAs) measured participant serum antibody concentrations (EU/mL) against the recombinant spike protein of the ancestral strain and Omicron BA.1 sublineage (previously validated [1] by Novavax Clinical Immunology [Gaithersburg, Maryland, USA]). This validated assay uses a full-length (1273 amino acid), wild-type ancestral SARS-CoV-2 S pre-fusion protein, with mutations in the furin cleavage site and two residues in the CH domain produced in Sf9 cells. The ELISA assay is quantitated to the WHO international standard (NIBSC code 20/136), which was previously described [20]. As an exploratory endpoint, pseudovirus neutralization assays provided 50% inhibitory dilution (ID_50_) data for the ancestral strain and Omicron BA.1 sublineage (validated by Monogram Biosciences [South San Francisco, CA, USA]). Pseudovirus assay was performed by Monogram Biosciences as described previously [18]. Neutralization titers were calculated as the inhibitory dilution (ID) of serum samples at which RLUs were reduced by either 50% (ID50) or 80% (ID80) compared to virus control wells (no serum wells).

Sample size determination (original target, n=360/group) was based on the co-primary endpoints of MN_50_ GMT and SRR. Study enrollment was halted ahead of reaching the planned population after re-examination of the sample size approximations suggested that the necessary number of participants had already been achieved (statistical power >85%) to assess the primary endpoints (new target, approximately n=235/group).

Randomization was managed by a contract research organization (CRO), and treatments were assigned using an Interactive Web Response System. The block size was blinded information and known only by the CRO statistician. Predetermined site personnel were unblinded to enable vaccine preparation and administration without breaking the blind for other personnel or participants. Only blinded personnel could perform study related assessments or have participant contact for data collection after administration of study vaccine.

**Reference**

1. Zhu M, Cloney-Clark S, Feng SL, et al. A Severe Acute Respiratory Syndrome Coronavirus 2 Anti-Spike Immunoglobulin G Assay: A Robust Method for Evaluation of Vaccine Immunogenicity Using an Established Correlate of Protection. Microorganisms. 2023 Jul 11;11(7).

**Supplemental Results**

***Participant Demographics***

The majority of participants in each group were female (52.2% [NVX-CoV2373, 143/274] to 56.1% [bivalent, 151/269]) and most participants were White (≥78.5% [NVX-CoV2515, 233/286; NVX-CoV2373, 215/274; bivalent, 220/269]) and of Australian ethnicity (≥86.1% [NVX-CoV2515, 252/286; NVX-CoV2373, 236/274; bivalent, 233/269]) (**Table 1**).

***Per Protocol Analysis Set 1:***

The ancestral strain anti-spike IgG assay reported GMEUs of 53,531.1 [46,055.5, 62,220.1], 40,423.4 [35,120.0, 46,527.7] and 42,783.9 [37,649.7, 48,618.1] EU/mL for NVX-CoV2373, NVX-CoV2515, and the bivalent vaccine, respectively (**Figure 1**).

***Per Protocol Analysis Set 2:***

The ancestral strain anti-spike IgG assay reported GMEUs of 90,962.2 [81,361.1, 101,696.2], 78,191.9 [69,489.1, 87,984.5], and 71,076.4 [63,012.1, 80,172.9] EU/mL for NVX-CoV2373, bivalent vaccine, and NVX-CoV2515, respectively (**Figure 2**).

**Supplemental Tables and Figures**

| **Supplemental Table 1. Demographics and baseline disease characteristics (PP1 and PP2 Analysis Sets)** | | | | | | |
| --- | --- | --- | --- | --- | --- | --- |
| **Parameters** | **PP1 Analysis Set** | | | **PP2 Analysis Set** | | |
|  | **NVX-CoV2515**  **N = 126** | **NVX-CoV2373**  **N = 119** | **Bivalent**  **(NVX‑CoV2373 +**  **NVX-CoV2515)**  **N = 118** | **NVX-CoV2515**  **N = 258** | **NVX-CoV2373**  **N = 251** | **Bivalent**  **(NVX‑CoV2373 +**  **NVX-CoV2515)**  **N = 240** |
| **Age (years)** | | | | | | |
| Mean (SD) | 42.0 (11.73) | 42.1 (11.10) | 42.0 (12.18) | 40.5 (12.4) | 40.0 (11.5) | 39.7 (12.5) |
| Median | 44.5 | 43.0 | 42.5 | 42.0 | 41.0 | 41.0 |
| Min–max | 20–64 | 18–63 | 18–63 | 18–64 | 18–64 | 18–64 |
| **Sex, n (%)**  SEX | | | | | | |
| Male | 47 (37.3) | 52 (43.7) | 52 (44.1) | 119 (46.1) | 122 (48.6) | 108 (45.0) |
| Female | 79 (62.7) | 67 (56.3) | 66 (55.9) | 139 (53.9) | 129 (51.4) | 132 (55.0) |
| **Race, n (%)**  RACE | | | | | | |
| White | 108 (85.7) | 98 (82.4) | 100 (84.7) | 208 (80.6) | 195 (77.7) | 195 (81.3) |
| Black or African American | 0 | 2 (1.7) | 0 | 0 | 2 (0.8) | 0 |
| Aboriginal Australian | 1 (0.8) | 0 | 0 | 2 (0.8) | 0 | 0 |
| Native Hawaiian or Other Pacific Islander | 0 | 0 | 1 (0.8) | 1 (0.4) | 0 | 1 (0.4) |
| Asian | 14 (11.1) | 15 (12.6) | 17 (14.4) | 35 (13.6) | 43 (17.1) | 37 (15.4) |
| Mixed Origin | 2 (1.6) | 3 (2.5) | 0 | 5 (1.9) | 3 (1.2) | 1 (0.4) |
| Other | 1 (0.8) | 1 (0.8) | 0 | 7 (2.7) | 8 (3.2) | 6 (2.5) |
| Not Reported | 0 | 0 | 0 | 0 | 0 | 0 |
| **Ethnicity, n (%)** | | | | | | |
| Australian | 116 (92.1) | 105 (88.2) | 108 (91.5) | 229 (88.8) | 217 (86.5) | 209 (87.1) |
| Aboriginal/Torres Strait Islanders | 1 (0.8) | 2 (1.7) | 0 | 4 (1.6) | 2 (0.8) | 0 |
| Hispanic or Latino | 1 (0.8) | 1 (0.8) | 1 (0.8) | 5 (1.9) | 7 (2.8) | 6 (2.5) |
| Not reported | 5 (4.0) | 5 (4.2) | 5 (4.2) | 9 (3.5) | 14 (5.6) | 14 (5.8) |
| Unknown | 3 (2.4) | 6 (5.0) | 3 (2.5) | 9 (3.5) | 10 (4.0) | 9 (3.8) |
| Missing | 0 | 0 | 1 (0.8) | 2 (0.8) | 1 (0.4) | 2 (0.8) |
| **BMI (kg/m^2^)** | | | | | | |
| Mean (SD) | 28.89 (6.900) | 28.06 (5.191) | 27.40 (5.841) | 28.2 (6.5) | 27.9 (5.2) | 27.4 (5.7) |
| Median | 27.40 | 27.50 | 26.20 | 27.20 | 27.50 | 26.30 |
| Min–max | 18.4–54.6 | 17.9–47.2 | 18.2–50.1 | 18.1–55.8 | 17.4–47.2 | 17.9–50.1 |
| **BMI (kg/m^2^) category, n (%)** | | | | | | |
| Underweight (<18.0) | 0 | 1 (0.8) | 0 | 0 | 3 (1.2) | 1 (0.4) |
| Normal (18.0–24.9) | 41 (32.5) | 32 (26.9) | 46 (39.0) | 93 (36.0) | 69 (27.5) | 94 (39.2) |
| Overweight (25.0–29.9) | 39 (31.0) | 49 (41.2) | 46 (39.0) | 80 (31.0) | 100 (39.8) | 82 (34.2) |
| Obese (≥ 30.0) | 46 (36.5) | 37 (31.1) | 26 (22.0) | 84 (32.6) | 76 (30.3) | 61 (25.4) |
| Missing | 0 | 0 | 0 | 1 (0.4) | 3 (1.2) | 2 (0.8) |
| **Regimen of previous COVID-19 vaccine, n (%)** | | | | | | |
| Moderna | 0 | 0 | 3 (2.5) | 0 | 2 (0.8) | 5 (2.1) |
| Pfizer-BioNTech | 95 (75.4) | 90 (75.6) | 89 (75.4) | 199 (77.1) | 194 (77.3) | 175 (72.9) |
| Mixed | 31 (24.6) | 29 (24.4) | 26 (22.0) | 59 (22.9) | 55 (21.9) | 60 (25.0) |
| Moderna-Moderna-Pfizer | 0 | 1 (0.8) | 0 | 1 (0.4) | 1 (0.4) | 0 |
| Moderna-Pfizer-Pfizer | 2 (1.6) | 0 | 0 | 2 (0.8) | 0 | 1 (0.4) |
| Moderna-Pfizer-Moderna | 0 | 0 | 0 | 0 | 0 | 0 |
| Pfizer-Pfizer-Moderna | 29 (23.0) | 28 (23.5) | 26 (22.0) | 56 (21.7) | 53 (21.1) | 59 (24.6) |
| Pfizer-Moderna-Moderna | 0 | 0 | 0 | 0 | 1 (0.4) | 0 |
| Pfizer-Moderna-Pfizer | 0 | 0 | 0 | 0 | 0 | 0 |
| **Previous COVID-19, n (%)** | | | | | | |
| Yes | 0 | 1 (0.8) | 1 (0.8) | 15 (5.8) | 16 (6.4) | 15 (6.3) |
| No | 126 (100) | 118 (99.2) | 117 (99.2) | 243 (94.2) | 235 (93.6) | 225 (93.8) |
| **Qualitative anti-N, n (%)** | | | | | | |
| Positive | 0 | 0 | 0 | 132 (51.2) | 132 (52.6) | 122 (50.8) |
| Negative | 126 (100) | 119 (100) | 118 (100) | 126 (48.8) | 119 (47.4) | 118 (49.2) |
| **PCR, n (%)** | | | | | | |
| Positive | 0 | 0 | 0 | 0 | 0 | 0 |
| Negative | 126 (100) | 119 (100) | 118 (100) | 258 (100) | 251 (100) | 240 (100) |
| **Anti-N / PCR, n (%)^1^** | | | | | | |
| Positive | 0 | 0 | 0 | 132 (51.2) | 132 (52.6) | 122 (50.8) |
| Negative | 126 (100) | 119 (100) | 118 (100) | 126 (48.8) | 119 (47.4) | 118 (49.2) |
| **Time between last previous COVID-19 vaccine and booster dose of study vaccine (days)** | | | | | | |
| Mean (SD) | 181.6 (39.09) | 181.5 (32.02) | 181.7 (32.24) | 177.7 (39.4) | 181.8 (35.9) | 178.8 (36.7) |
| Median | 178.0 | 181.0 | 182.5 | 177.0 | 182.0 | 180.0 |
| Min–max | 105–440 | 91–267 | 110–306 | 84–440 | 91–329 | 91–313 |
| **Interval between last previous COVID-19 vaccine and booster dose of study vaccine, n (%)** | | | | | | |
| <90 days | 0 | 0 | 0 | 1 (0.4) | 0 | 0 |
| 90–120 days | 4 (3.2) | 4 (3.4) | 4 (3.4) | 15 (5.8) | 15 (6.0) | 17 (7.1) |
| >120–150 days | 17 (13.5) | 16 (13.4) | 15 (12.7) | 38 (14.7) | 31 (12.4) | 33 (13.8) |
| >150–180 days | 49 (38.9) | 38 (31.9) | 36 (30.5) | 87 (33.7) | 73 (29.1) | 72 (30.0) |
| >180–210 days | 38 (30.2) | 43 (36.1) | 46 (39.0) | 80 (31.0) | 89 (35.5) | 82 (34.2) |
| >210–240 days | 10 (7.9) | 13 (10.9) | 12 (10.2) | 23 (8.9) | 31 (12.4) | 24 (10.0) |
| >240–270 days | 5 (4.0) | 5 (4.2) | 4 (3.4) | 8 (3.1) | 9 (3.6) | 9 (3.8) |
| >270–300 days | 2 (1.6) | 0 | 0 | 4 (1.6) | 1 (0.4) | 1 (0.4) |
| >300–330 days | 0 | 0 | 1 (0.8) | 1 (0.4) | 2 (0.8) | 2 (0.8) |
| >330–360 days | 0 | 0 | 0 | 0 | 0 | 0 |
| >360 days | 1 (0.8) | 0 | 0 | 1 (0.4) | 0 | 0 |
| Abbreviations: anti-N = anti-nucleocapsid; BMI = body mass index; COVID-19 = coronavirus disease 2019; max = maximum; min = minimum; NVX‑CoV2515 = 5 µg SARS‑CoV‑2 rS with 50 µg Matrix-M^TM^ adjuvant; NVX‑CoV2373 = 5 µg SARS‑CoV‑2 rS with 50 µg Matrix-M adjuvant; NVX‑CoV2373 + NVX-CoV2515 = 5 µg SARS‑CoV‑2 rS with 50 µg Matrix-M adjuvant (total); PCR = polymerase chain reaction; SARS-CoV-2 = severe acute respiratory syndrome coronavirus 2; SARS-CoV-2 rS = severe acute respiratory syndrome coronavirus 2 recombinant spike protein nanoparticle vaccine; SD = standard deviation.  Note: Age was calculated at the time of informed consent.  Note: n for continuous parameters represents the number of participants with non-missing values for that parameter.  Note: BMI was calculated as weight (kg) divided by squared height (m). Percentages were based on the respective Per Protocol Analysis Set within each treatment and overall.  1. Participants with either anti-N or PCR are reported. | | | | | | |

**Supplemental Figure 1. CONSORT diagram**

^
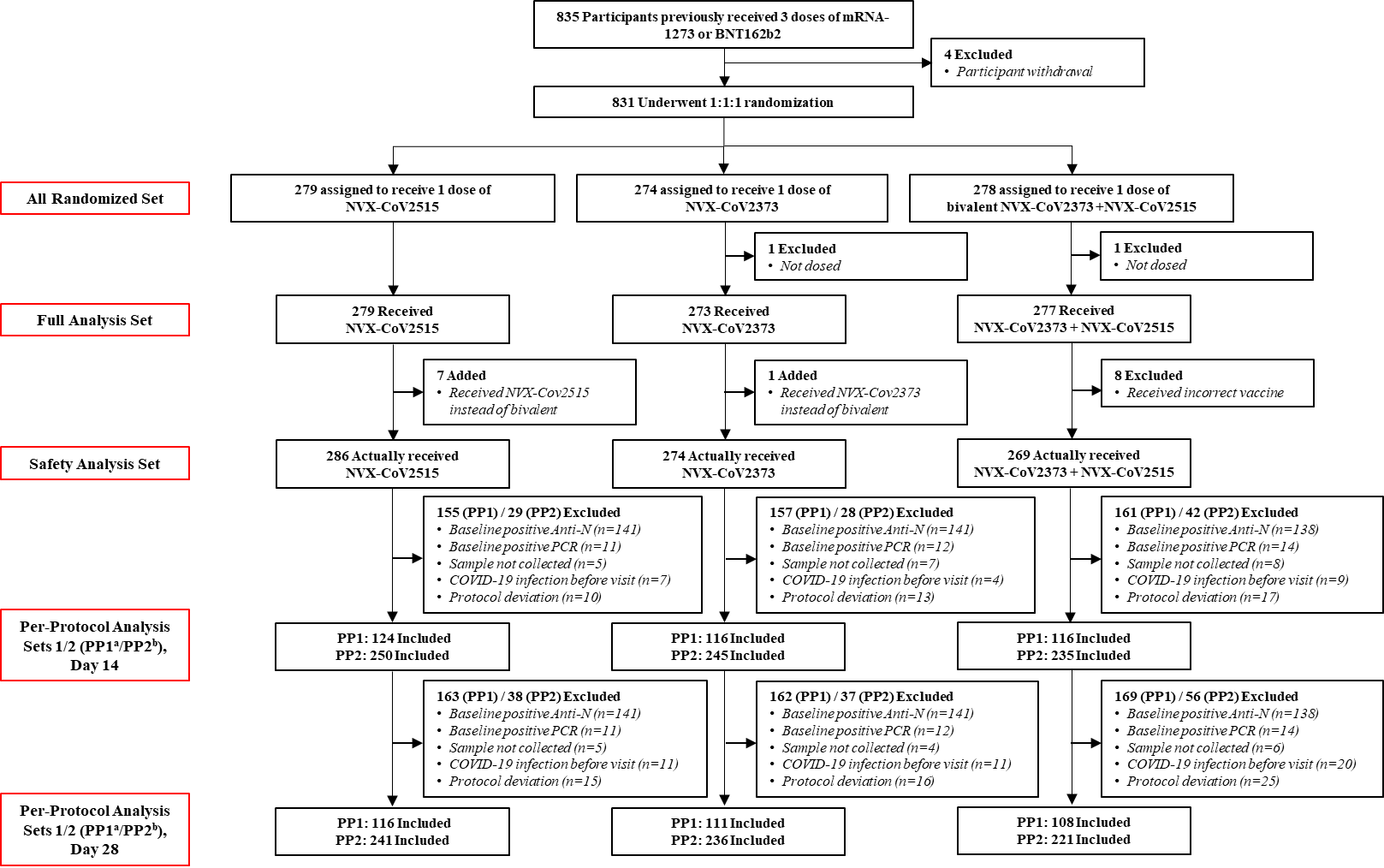
^
^a^PP1 includes all participants who received the full prescribed regimen of the study vaccine, had serology results for baseline and the time point analyzed, were negative at baseline for SARS-CoV-2, and had no major protocol violations or an event (e.g., COVID-19 infection) that was considered clinically relevant to impact immunogenicity response. ^b^PP2 population defined exactly as the PP1 population except that participants positive at baseline for SARS-CoV-2 were included.

| \| **Supplemental Table 2. Serum microneutralization titers, anti-rS IgG antibody concentrations, and pseudovirus neutralization titers against the Ancestral and Omicron BA.1 variant strain following a heterologous 4^th^ booster dose (PP1 Analysis Set)** \| \| \| \| \| \| \| \| --- \| --- \| --- \| --- \| --- \| --- \| --- \| \| **Vaccine Group** \| **NVX-CoV2515 (5 ug Omicron BA.1 rS)**  **N = 126** \| \| **NVX-CoV2373 (5 ug ancestral rS)**  **N = 119** \| \| **Bivalent (2.5 ug ancestral rS + 2.5 ug Omicron BA.1 rS)**  **N = 118** \| \| \| **Ancestral** \| **BA.1** \| **Ancestral** \| **BA.1** \| **Ancestral** \| **BA.1** \| \| **Microneutralization titers (MN_50_)** \| \| \| \| \| \| \| \| **Day 0 (baseline)^a^** \| \| \| \| \| \| \| \| n1 (all participants with non-missing data) \| 126 \| 126 \| 119 \| 119 \| 114 \| 114 \| \| Median \| 640.0 \| 20.0 \| 640.0 \| 20.0 \| 480.0 \| 20.0 \| \| Min, max \| 20–5120 \| 10–320 \| 20–10240 \| 10–1280 \| 40–5120 \| 10–320 \| \| GMT (MN_50_) \| 457.6 \| 25.2 \| 486.7 \| 27.9 \| 431.1 \| 26.0 \| \| 95% CI^b^ \| 378.2, 553.5 \| 21.5, 29.5 \| 393.3, 602.3 \| 22.9, 33.9 \| 359.4, 517.0 \| 21.8, 30.9 \| \| **Day 14** \| \| \| \| \| \| \| \| n1 \| 124 \| 124 \| 116 \| 116 \| 113 \| 113 \| \| Median \| 1280.0 \| 160.0 \| 1280.0 \| 80.0 \| 1280.0 \| 80.0 \| \| Min, max \| 80–10240 \| 10–1280 \| 160–40960 \| 10–1280 \| 80–10240 \| 10–1280 \| \| GMT (MN_50_) \| 1076.3 \| 130.8 \| 1442.5 \| 83.9 \| 1319.9 \| 97.9 \| \| 95% CI^b^ \| 908.4, 1275.4 \| 109.2, 156.7 \| 1192.4, 1745.0 \| 69.6, 101.2 \| 1120.1, 1555.3 \| 81.3, 117.9 \| \| n2 (participants with non-missing data at Day 0 and Day 14) \| 124 \| 124 \| 116 \| 116 \| 113 \| 113 \| \| GMFR referencing Day 0 \| 2.4 \| 5.2 \| 3.0 \| 3.0 \| 3.1 \| 3.7 \| \| 95% CI^b^ \| 2.0, 2.8 \| 4.4, 6.1 \| 2.5, 3.5 \| 2.6, 3.6 \| 2.6, 3.6 \| 3.2, 4.4 \| \| SRR ≥4-fold increase,^c^ n3 (participants with ≥4-fold increase) /n2 (%) \| 54/124 (43.5) \| 91/124 (73.4) \| 57/116 (49.1) \| 59/116 (50.9) \| 64/113 (56.6) \| 74/113 (65.5) \| \| 95% CI^d^ \| 34.7, 52.7 \| 64.7, 80.9 \| 39.7, 58.6 \| 41.4, 60.3 \| 47.0, 65.9 \| 56.0, 74.2 \| \| **Day 28** \| \| \| \| \| \| \| \| n1 \| 116 \| 116 \| 110 \| 110 \| 102 \| 102 \| \| Median \| 1280.0 \| 160.0 \| 1280.0 \| 80.0 \| 1280.0 \| 80.0 \| \| Min, max \| 40–10240 \| 10–2560 \| 80–40960 \| 10–2560 \| 160–10240 \| 10–640 \| \| GMT (MN_50_) \| 960.8 \| 122.3 \| 1442.8 \| 77.5 \| 1087.4 \| 87.4 \| \| 95% CI^b^ \| 798.0, 1156.9 \| 101.0, 148.0 \| 1188.1, 1752.1 \| 63.1, 95.3 \| 906.5, 1304.3 \| 72.0, 106.1 \| \| n2 (participants with non-missing data at Day 0 and Day 28) \| 116 \| 116 \| 110 \| 110 \| 102 \| 102 \| \| GMFR referencing  Day 0 \| 2.0 \| 4.8 \| 3.0 \| 2.9 \| 2.4 \| 3.3 \| \| 95% CI^b^ \| 1.7, 2.4 \| 4.1, 5.7 \| 2.5, 3.6 \| 2.4, 3.6 \| 2.1, 2.8 \| 2.8, 3.8 \| \| SRR ≥4-fold increase,^c^ n3/n2 (%) \| 37/116 (31.9) \| 86/116 (74.1) \| 54/110 (49.1) \| 52/110 (47.3) \| 42/102 (41.2) \| 56/102 (54.9) \| \| 95% CI^d^ \| 23.6, 41.2 \| 65.2, 81.8 \| 39.4, 58.8 \| 37.7, 57.0 \| 31.5, 51.4 \| 44.7, 64.8 \| \| **Serum anti-rS IgG antibodies (EU/mL)** \| \| \| \| \| \| \| \| **Day 0 (baseline)^a^** \| \| \| \| \| \| \| \| n1 \| 126 \| 126 \| 118 \| 118 \| 118 \| 118 \| \| Median \| 18549.0 \| 9709.0 \| 18415.0 \| 8654.0 \| 16869.5 \| 9021.5 \| \| Min, max \| 2915–146257 \| 652–64111 \| 2232–1002823 \| 864–249167 \| 2279–212765 \| 814–117381 \| \| GMEU (EU/mL) \| 18773.6 \| 9117.3 \| 20168.6 \| 9770.2 \| 17803.1 \| 8598.7 \| \| 95% CI^e^ \| 16206.3, 21747.5 \| 7796.7, 10661.7 \| 16760.8, 24269.2 \| 8107.6, 11773.8 \| 15264.7, 20763.6 \| 7326.9, 10091.1 \| \| **Day 14** \| \| \| \| \| \| \| \| n1 \| 124 \| 124 \| 115 \| 115 \| 116 \| 116 \| \| Median \| 42975.0 \| 26627.5 \| 55183.0 \| 32732.0 \| 40265.0 \| 23036.0 \| \| Min, max \| 3973–399697 \| 1623–234270 \| 5604–518336 \| 2097–358950 \| 6811–232384 \| 3178–233435 \| \| GMEU (EU/mL) \| 40423.4 \| 24174.8 \| 53531.1 \| 30170.9 \| 42783.9 \| 23045.5 \| \| 95% CI^e^ \| 35120.0, 46527.7 \| 20943.6, 27904.6 \| 46055.5, 62220.1 \| 25663.7, 35469.6 \| 37649.7, 48618.1 \| 20113.5, 26404.8 \| \| n2 \| 124 \| 124 \| 115 \| 115 \| 116 \| 116 \| \| GMFR referencing Day 0 \| 2.2 \| 2.7 \| 2.7 \| 3.1 \| 2.4 \| 2.7 \| \| 95% CI^e^ \| 1.9, 2.4 \| 2.4, 3.0 \| 2.3, 3.0 \| 2.7, 3.5 \| 2.1, 2.7 \| 2.4, 3.0 \| \| SRR ≥4-fold increase,^f^ n3/n2 (%) \| 17/124 (13.7) \| 31/124 (25.0) \| 31/115 (27.0) \| 41/115 (35.7) \| 23/116 (19.8) \| 29/116 (25.0) \| \| 95% CI^d^ \| 8.2, 21.0 \| 17.7, 33.6 \| 19.1, 36.0 \| 26.9, 45.1 \| 13.0, 28.3 \| 17.4, 33.9 \| \| **Day 28** \| \| \| \| \| \| \| \| n1 \| 116 \| 116 \| 110 \| 110 \| 108 \| 108 \| \| Median \| 41916.5 \| 25590.5 \| 56963.5 \| 32178.0 \| 39302.5 \| 21025.0 \| \| Min, max \| 3572–338495 \| 1595–183367 \| 5668–675805 \| 1606–361845 \| 6763–340211 \| 2699–256572 \| \| GMEU (EU/mL) \| 40833.4 \| 22732.1 \| 54598.2 \| 28448.6 \| 41070.0 \| 21772.1 \| \| 95% CI^e^ \| 35196.6, 47373.1 \| 19381.6, 26661.8 \| 46668.3, 63875.5 \| 24062.8, 33633.8 \| 35625.2, 47346.9 \| 18753.6, 25276.5 \| \| n2 \| 116 \| 116 \| 110 \| 110 \| 108 \| 108 \| \| GMFR referencing Day 0 \| 2.1 \| 2.5 \| 2.8 \| 3.0 \| 2.3 \| 2.5 \| \| 95% CI^e^ \| 1.9, 2.4 \| 2.2, 2.8 \| 2.4, 3.2 \| 2.6, 3.4 \| 2.0, 2.5 \| 2.2, 2.8 \| \| SRR ≥4-fold increase,^f^ n3/n2 (%) \| 19/116 (16.4) \| 27/116 (23.3) \| 33/110 (30.0) \| 35/110 (31.8) \| 17/108 (15.7) \| 22/108 (20.4) \| \| 95% CI^d^ \| 10.2, 24.4 \| 15.9, 32.0 \| 21.6, 39.5 \| 23.3, 41.4 \| 9.4, 24.0 \| 13.2, 29.2 \| \| **Pseudovirus neutralization titers (ID_50_)** \| \| \| \| \| \| \| \| **Day 0 (baseline)^a^** \| \| \| \| \| \| \| \| n1 \| 111 \| 111 \| 105 \| 105 \| 105 \| 105 \| \| Median \| 584.0 \| 116.0 \| 431.0 \| 124.0 \| 476.0 \| 96.0 \| \| Min, max \| 20–4746 \| 20–2937 \| 20–26040 \| 20–7900 \| 50–7541 \| 20–5317 \| \| GMT (ID_50_) \| 496.7 \| 99.6 \| 517.1 \| 103.6 \| 483.3 \| 89.2 \| \| 95% CI^b^ \| 401.0, 615.2 \| 77.1, 128.8 \| 409.3, 653.1 \| 77.9, 137.8 \| 400.7, 583.0 \| 68.2, 116.9 \| \| **Day 14** \| \| \| \| \| \| \| \| n1 \| 110 \| 110 \| 102 \| 102 \| 103 \| 103 \| \| Median \| 1341.0 \| 930.5 \| 1476.0 \| 433.5 \| 1084.0 \| 496.0 \| \| Min, max \| 20–19256 \| 20–30281 \| 273–34712 \| 20–15337 \| 153–11739 \| 20–6949 \| \| GMT (ID_50_) \| 1181.1 \| 816.1 \| 1473.0 \| 449.1 \| 1195.1 \| 562.0 \| \| 95% CI^b^ \| 976.6, 1428.3 \| 639.9, 1040.7 \| 1227.1, 1768.3 \| 345.3, 584.2 \| 1012.1, 1411.2 \| 439.6, 718.6 \| \| n2 \| 110 \| 110 \| 102 \| 102 \| 103 \| 103 \| \| GMFR referencing Day 0 \| 2.4 \| 8.2 \| 2.9 \| 4.4 \| 2.5 \| 6.3 \| \| 95% CI^b^ \| 2.0, 2.8 \| 6.5, 10.5 \| 2.5, 3.4 \| 3.6, 5.5 \| 2.2, 2.8 \| 5.0, 7.9 \| \| SRR ≥4-fold increase,^c^ n3/n2 (%) \| 21/110 (19.1) \| 81/110 (73.6) \| 25/102 (24.5) \| 48/102 (47.1) \| 25/103 (24.3) \| 68/103 (66.0) \| \| 95% CI^d^ \| 12.2, 27.7 \| 64.4, 81.6 \| 16.5, 34.0 \| 37.1, 57.2 \| 16.4, 33.7 \| 56.0, 75.1 \| \| **Day 28** \| \| \| \| \| \| \| \| n1 \| 101 \| 101 \| 98 \| 98 \| 97 \| 97 \| \| Median \| 1219.0 \| 953.0 \| 1390.0 \| 425.5 \| 1043.0 \| 502.0 \| \| Min, max \| 20–15355 \| 20–9668 \| 20–26603 \| 20–25073 \| 154–9349 \| 20–5474 \| \| GMT (ID_50_) \| 1085.8 \| 661.6 \| 1420.2 \| 404.1 \| 1106.1 \| 502.1 \| \| 95% CI^b^ \| 891.5, 1322.4 \| 509.7, 858.7 \| 1141.9, 1766.2 \| 305.4, 534.7 \| 937.7, 1304.8 \| 393.7, 640.4 \| \| n2 \| 101 \| 101 \| 98 \| 98 \| 97 \| 97 \| \| GMFR referencing Day 0 \| 2.2 \| 6.7 \| 2.9 \| 4.1 \| 2.3 \| 5.6 \| \| 95% CI^b^ \| 1.9, 2.6 \| 5.2, 8.7 \| 2.3, 3.5 \| 3.2, 5.3 \| 2.0, 2.6 \| 4.4, 7.0 \| \| SRR ≥4-fold increase,^c^ n3/n2 (%) \| 20/101 (19.8) \| 69/101 (68.3) \| 29/98 (29.6) \| 46/98 (46.9) \| 18/97 (18.6) \| 58/97 (59.8) \| \| 95% CI^d^ \| 12.5, 28.9 \| 58.3, 77.2 \| 20.8, 39.7 \| 36.8, 57.3 \| 11.4, 27.7 \| 49.3, 69.6 \|   Abbreviations: CI = confidence interval; GMEU= geometric mean ELISA units; GMFR = geometric mean fold rise; GMT = geometric mean titer; ID_50_ = inhibitory dilution with a 50% concentration; Max = maximum; Min = minimum; N = number of participants in the assay-specific analysis Set; n1 = number of participants within each visit with non-missing data; n2 = number of participants with non-missing data at both visits of interest; n3 = number of participants who reported ≥4-fold increase with percentages calculated based on n2 as the denominator; LLOQ = lower limit of quantitation; SRR = seroresponse rate.  ^a^Baseline was defined as the last non-missing assessment prior to first vaccination. ^b^The 95% CI for GMT and GMFR were calculated based on the t-distribution of the log-transformed values then back transformed to the original scale for presentation. ^c^The SRR was defined as percentage of participants at each post vaccination visit with a titer ≥4-fold rise in MN_50_ or ID_50_ level. ^d^The 95% CI for SRR was calculated using the exact Clopper-Pearson method. ^e^The 95% CI for GMEU and GMFR were calculated based on the t-distribution of the log-transformed values then back transformed to the original scale for presentation. ^f^The SRR was defined as percentage of participants at each post vaccination visit with a titer ≥4-fold rise in anti-S IgG antibody level.  Note: Values less than LLOQ were replaced by 0.5 × LLOQ |
| --- | --- | --- | --- | --- | --- | --- | --- | --- | --- | --- | --- | --- | --- | --- | --- | --- | --- | --- | --- | --- | --- | --- | --- | --- | --- | --- | --- | --- | --- | --- | --- | --- | --- | --- | --- | --- | --- | --- | --- | --- | --- | --- | --- | --- | --- | --- | --- | --- | --- | --- | --- | --- | --- | --- | --- | --- | --- | --- | --- | --- | --- | --- | --- | --- | --- | --- | --- | --- | --- | --- | --- | --- | --- | --- | --- | --- | --- | --- | --- | --- | --- | --- | --- | --- | --- | --- | --- | --- | --- | --- | --- | --- | --- | --- | --- | --- | --- | --- | --- | --- | --- | --- | --- | --- | --- | --- | --- | --- | --- | --- | --- | --- | --- | --- | --- | --- | --- | --- | --- | --- | --- | --- | --- | --- | --- | --- | --- | --- | --- | --- | --- | --- | --- | --- | --- | --- | --- | --- | --- | --- | --- | --- | --- | --- | --- | --- | --- | --- | --- | --- | --- | --- | --- | --- | --- | --- | --- | --- | --- | --- | --- | --- | --- | --- | --- | --- | --- | --- | --- | --- | --- | --- | --- | --- | --- | --- | --- | --- | --- | --- | --- | --- | --- | --- | --- | --- | --- | --- | --- | --- | --- | --- | --- | --- | --- | --- | --- | --- | --- | --- | --- | --- | --- | --- | --- | --- | --- | --- | --- | --- | --- | --- | --- | --- | --- | --- | --- | --- | --- | --- | --- | --- | --- | --- | --- | --- | --- | --- | --- | --- | --- | --- | --- | --- | --- | --- | --- | --- | --- | --- | --- | --- | --- | --- | --- | --- | --- | --- | --- | --- | --- | --- | --- | --- | --- | --- | --- | --- | --- | --- | --- | --- | --- | --- | --- | --- | --- | --- | --- | --- | --- | --- | --- | --- | --- | --- | --- | --- | --- | --- | --- | --- | --- | --- | --- | --- | --- | --- | --- | --- | --- | --- | --- | --- | --- | --- | --- | --- | --- | --- | --- | --- | --- | --- | --- | --- | --- | --- | --- | --- | --- | --- | --- | --- | --- | --- | --- | --- | --- | --- | --- | --- | --- | --- | --- | --- | --- | --- | --- | --- | --- | --- | --- | --- | --- | --- | --- | --- | --- | --- | --- | --- | --- | --- | --- | --- | --- | --- | --- | --- | --- | --- | --- | --- | --- | --- | --- | --- | --- | --- | --- | --- | --- | --- | --- | --- | --- | --- | --- | --- | --- | --- | --- | --- | --- | --- | --- | --- | --- | --- | --- | --- | --- | --- | --- | --- | --- | --- | --- | --- | --- | --- | --- | --- | --- | --- | --- | --- | --- | --- | --- | --- | --- | --- | --- | --- | --- | --- | --- | --- | --- | --- | --- | --- | --- | --- | --- | --- | --- | --- | --- | --- | --- | --- | --- | --- | --- | --- | --- | --- | --- | --- | --- | --- | --- | --- | --- | --- | --- | --- | --- | --- | --- | --- | --- | --- | --- | --- | --- | --- | --- | --- | --- | --- | --- | --- | --- | --- | --- | --- | --- | --- | --- | --- | --- | --- | --- | --- | --- | --- | --- | --- | --- | --- | --- | --- | --- | --- | --- | --- | --- | --- | --- | --- | --- | --- | --- | --- | --- | --- | --- | --- | --- | --- | --- | --- | --- | --- | --- | --- | --- | --- | --- | --- | --- | --- | --- | --- | --- | --- | --- | --- | --- | --- | --- | --- | --- | --- | --- | --- | --- | --- | --- | --- | --- | --- | --- | --- | --- | --- | --- | --- | --- | --- | --- | --- | --- | --- | --- | --- | --- | --- | --- | --- | --- | --- | --- | --- | --- | --- | --- | --- | --- | --- | --- | --- | --- | --- | --- | --- | --- | --- | --- | --- | --- | --- | --- | --- | --- | --- | --- | --- | --- | --- | --- | --- | --- | --- | --- | --- | --- | --- | --- | --- | --- | --- | --- | --- | --- | --- | --- | --- | --- | --- | --- | --- | --- | --- | --- | --- | --- | --- | --- | --- | --- | --- | --- | --- | --- | --- | --- | --- | --- | --- | --- | --- | --- | --- | --- | --- | --- | --- | --- | --- | --- | --- | --- | --- | --- |

| **Supplemental Table 3. Serum microneutralization titers, anti-rS IgG antibody concentrations, and pseudovirus neutralization titers against the Ancestral and Omicron BA.1 variant strain following a heterologous 4^th^ booster dose (PP2 Analysis Set)** | | | | | | | | |
| --- | --- | --- | --- | --- | --- | --- | --- | --- |
| **Vaccine Group** | **NVX-CoV2515**  **N = 286** | | | **NVX-CoV2373**  **N = 274** | | | **Bivalent**  **N = 269** | |
|  | **Ancestral** | **BA.1** | **Ancestral** | | **BA.1** | **Ancestral** | | **BA.1** |
| **Microneutralization titers (MN_50_)** | | | | | | | | |
| **Day 0 (baseline)^a^** | | | | | | | | |
| n1 (all participants with non-missing data) | 255 | 255 | 250 | | 250 | 236 | | 236 |
| Median | 1280.0 | 160.0 | 1280.0 | | 160.0 | 1280.0 | | 160.0 |
| Min–max | 20–40960 | 10–2560 | 20–40960 | | 10–2560 | 40–81920 | | 10–10240 |
| GMT (MN_50_) | 1151.3 | 97.3 | 1280.0 | | 105.9 | 1232.0 | | 106.1 |
| 95% CI^b^ | 973.6, 1361.4 | 79.6, 118.9 | 1078.1, 1519.7 | | 86.4, 129.7 | 1026.0, 1479.5 | | 85.8, 131.1 |
| **Day 14** | | | | | | | | |
| n1 | 247 | 247 | 244 | | 244 | 232 | | 232 |
| Median | 2560.0 | 320.0 | 2560.0 | | 320.0 | 2560.0 | | 320.0 |
| Min–max | 80–40960 | 10–5120 | 160–81920 | | 10–2560 | 80–163840 | | 10–5120 |
| GMT (MN_50_) | 2206.2 | 318.2 | 2702.0 | | 218.1 | 2544.7 | | 252.7 |
| 95% CI^b^ | 1910.0, 2548.4 | 269.8, 375.3 | 2347.9, 3109.4 | | 186.0, 255.7 | 2194.5, 2950.9 | | 213.1, 299.7 |
| n2 (participants with non-missing data at Day 0 and Day 14) | 247 | 247 | 244 | | 244 | 232 | | 232 |
| GMFR referencing Day 0 | 1.9 | 3.3 | 2.1 | | 2.1 | 2.1 | | 2.4 |
| 95% CI^b^ | 1.8, 2.1 | 2.9, 3.7 | 1.9, 2.4 | | 1.8, 2.3 | 1.9, 2.3 | | 2.1, 2.7 |
| SRR ≥4-fold increase,^c^  n3 (participants with ≥4-fold increase) /n2 (%) | 79/247 (32.0) | 134/247 (54.3) | 80/244 (32.8) | | 78/244 (32.0) | 83/232 (35.8) | | 95/232 (40.9) |
| 95% CI^d^ | 26.2, 38.2 | 47.8, 60.6 | 26.9, 39.1 | | 26.2, 38.2 | 29.6, 42.3 | | 34.6, 47.6 |
| **Day 28** | | | | | | | | |
| n1 | 238 | 238 | 234 | | 234 | 215 | | 215 |
| Median | 2560.0 | 320.0 | 2560.0 | | 160.0 | 2560.0 | | 160.0 |
| Min–max | 40–20480 | 10–5120 | 80–40960 | | 10–2560 | 160–81920 | | 10–10240 |
| GMT (MN_50_) | 1918.8 | 284.8 | 2456.0 | | 195.7 | 2144.0 | | 218.7 |
| 95% CI^b^ | 1657.9, 2220.6 | 241.8, 335.4 | 2145.2, 2811.8 | | 165.7, 231.2 | 1842.3, 2495.2 | | 183.1, 261.3 |
| n2 (participants with non-missing data at Day 0 and Day 28) | 238 | 238 | 234 | | 234 | 215 | | 215 |
| GMFR referencing Day 0 | 1.6 | 2.9 | 1.9 | | 1.9 | 1.7 | | 2.0 |
| 95% CI^b^ | 1.5, 1.8 | 2.5, 3.3 | 1.7, 2.2 | | 1.6, 2.2 | 1.5, 1.9 | | 1.8, 2.2 |
| SRR ≥4-fold increase,^c^  n3/n2 (%) | 56/238 (23.5) | 125/238 (52.5) | 68/234 (29.1) | | 65/234 (27.8) | 59/215 (27.4) | | 72/215 (33.5) |
| 95% CI^d^ | 18.3, 29.4 | 46.0, 59.0 | 23.3, 35.3 | | 22.1, 34.0 | 21.6, 33.9 | | 27.2, 40.2 |
| **Serum anti-rS IgG antibodies (EU/mL)** | | | | | | | | |
| **Day 0 (baseline)^a^** | | | | | | | | |
| n1 | 258 | 258 | 250 | | 250 | 240 | | 240 |
| Median | 45426.5 | 22876.0 | 54982.0 | | 28832.0 | 49528.0 | | 25602.0 |
| Min–max | 2915–576831 | 652–353367 | 2232–1002823 | | 864–254105 | 2279–2034472 | | 814–1121904 |
| GMEU (EU/mL) | 44166.7 | 21446.9 | 47810.6 | | 23215.7 | 45047.6 | | 22400.7 |
| 95% CI^e^ | 38369.6, 50839.6 | 18570.6, 24768.7 | 41184.3, 55503.1 | | 19953.4, 27011.3 | 38572.8, 52609.1 | | 19088.9, 26287.2 |
| **Day 14** | | | | | | | | |
| n1 | 250 | 250 | 244 | | 244 | 235 | | 235 |
| Median | 72765.5 | 41964.5 | 95097.0 | | 50675.0 | 79657.0 | | 43289.0 |
| Min–max | 3973–894339 | 1623–813679 | 5604–822744 | | 2097–395875 | 6811–1656933 | | 3178–1074179 |
| GMEU (EU/mL) | 71076.4 | 42835.5 | 90962.2 | | 49727.7 | 78191.9 | | 42462.1 |
| 95% CI^e^ | 63012.1, 80172.9 | 37883.8, 48434.4 | 81361.1, 101696.2 | | 44331.1, 55781.1 | 69489.1, 87984.5 | | 37628.9, 47916.2 |
| n2 | 250 | 250 | 244 | | 244 | 235 | | 235 |
| GMFR referencing Day 0 | 1.6 | 2.0 | 1.9 | | 2.1 | 1.7 | | 1.9 |
| 95% CI^e^ | 1.5, 1.7 | 1.8, 2.2 | 1.8, 2.1 | | 2.0, 2.3 | 1.6, 1.9 | | 1.7, 2.0 |
| SRR ≥4-fold increase,^f^  n3/n2 (%) | 18/250 (7.2) | 34/250 (13.6) | 35/244 (14.3) | | 42/244 (17.2) | 23/235 (9.8) | | 29/235 (12.3) |
| 95% CI^d^ | 4.3, 11.1 | 9.6, 18.5 | 10.2, 19.4 | | 12.7, 22.5 | 6.3, 14.3 | | 8.4, 17.2 |
| **Day 28** | | | | | | | | |
| n1 | 241 | 241 | 235 | | 235 | 221 | | 221 |
| Median | 72699.0 | 42388.0 | 92903.0 | | 44971.0 | 70184.0 | | 36642.0 |
| Min–max | 3572–755671 | 1595–543014 | 5668–675805 | | 1606–375634 | 6763–2299922 | | 2699–1217244 |
| GMC (EU/mL) | 70523.9 | 40044.9 | 87969.9 | | 44756.4 | 73321.3 | | 38315.6 |
| 95% CI^e^ | 62487.3, 79594.2 | 35308.6, 45416.6 | 78701.3, 98330.0 | | 39966.0, 50120.9 | 64771.3, 83000.0 | | 33783.4, 43455.9 |
| n2 | 241 | 241 | 235 | | 235 | 221 | | 221 |
| GMFR referencing Day 0 | 1.6 | 1.8 | 1.9 | | 2.0 | 1.6 | | 1.7 |
| 95% CI^e^ | 1.4, 1.7 | 1.7, 2.0 | 1.7, 2.0 | | 1.8, 2.1 | 1.5, 1.8 | | 1.6, 1.8 |
| SRR ≥4-fold increase,^f^  n3/n2 (%) | 19/241 (7.9) | 28/241 (11.6) | 34/235 (14.5) | | 37/235 (15.7) | 17/221 (7.7) | | 22/221 (10.0) |
| 95% CI^d^ | 4.8, 12.0 | 7.9, 16.4 | 10.2, 19.6 | | 11.3, 21.0 | 4.5, 12.0 | | 6.3, 14.7 |
| **Pseudovirus neutralization titers (ID_50_)** | | | | | | | | |
| **Day 0 (baseline)^a^** | | | | | | | | |
| n1 | 232 | 232 | 226 | | 226 | 206 | | 206 |
| Median | 1788.5 | 808.0 | 2224.5 | | 962.0 | 1425.5 | | 623.5 |
| Min, max | 20–39721 | 20–30397 | 20–28737 | | 20–18494 | 50–81399 | | 20–84031 |
| GMT (ID_50_) | 1491.2 | 548.4 | 1646.5 | | 579.2 | 1476.4 | | 491.9 |
| 95% CI^b^ | 1237.3, 1797.1 | 421.7, 713.2 | 1357.6, 1996.8 | | 445.6, 752.8 | 1208.2, 1804.1 | | 366.8, 659.6 |
| **Day 14** | | | | | | | | |
| n1 | 223 | 223 | 220 | | 220 | 201 | | 201 |
| Median | 2464.0 | 2202.0 | 3121.5 | | 1690.5 | 2526.0 | | 1677.0 |
| Min, max | 20–34131 | 20–41483 | 273–34859 | | 20–23694 | 153–71353 | | 20–72247 |
| GMT (ID_50_) | 2514.9 | 2071.5 | 3027.9 | | 1390.3 | 2390.1 | | 1480.4 |
| 95% CI^b^ | 2149.8, 2942.0 | 1701.1, 2522.5 | 2625.7, 3491.6 | | 1134.6, 1703.8 | 2050.2, 2786.3 | | 1202.8, 1822.1 |
| n2 | 223 | 223 | 220 | | 220 | 201 | | 201 |
| GMFR referencing Day 0 | 1.7 | 3.9 | 1.8 | | 2.4 | 1.6 | | 3.0 |
| 95% CI^b^ | 1.5, 1.9 | 3.3, 4.6 | 1.7, 2.0 | | 2.1, 2.8 | 1.5, 1.8 | | 2.5, 3.5 |
| SRR ≥4-fold increase,^c^ n3/n2 (%) | 25/223 (11.2) | 102/223 (45.7) | 26/220 (11.8) | | 53/220 (24.1) | 25/201 (12.4) | | 71/201 (35.3) |
| 95% CI^d^ | 7.4, 16.1 | 39.1, 52.5 | 7.9, 16.8 | | 18.6, 30.3 | 8.2, 17.8 | | 28.7, 42.4 |
| **Day 28** | | | | | | | | |
| n1 | 214 | 214 | 212 | | 212 | 191 | | 191 |
| Median | 2445.0 | 2127.5 | 2875.0 | | 1332.5 | 2193.0 | | 1303.0 |
| Min, max | 20–45309 | 20–70894 | 20–31723 | | 20–25073 | 154–168338 | | 20–95088 |
| GMT (ID_50_) | 2411.6 | 1918.0 | 2716.9 | | 1195.7 | 2236.0 | | 1272.4 |
| 95% CI^b^ | 2055.6, 2829.2 | 1558.2, 2361.0 | 2340.0, 3154.5 | | 974.9, 1466.6 | 1909.8, 2617.9 | | 1033.5, 1566.6 |
| n2 | 214 | 214 | 212 | | 212 | 191 | | 191 |
| GMFR referencing Day 0 | 1.6 | 3.4 | 1.7 | | 2.1 | 1.5 | | 2.6 |
| 95% CI^b^ | 1.5, 1.8 | 2.9, 4.0 | 1.5, 1.9 | | 1.8, 2.5 | 1.4, 1.7 | | 2.2, 3.0 |
| SRR ≥4-fold increase,^c^ n3/n2 (%) | 22/214 (10.3) | 84/214 (39.3) | 29/212 (13.7) | | 48/212 (22.6) | 19/191 (9.9) | | 61/191 (31.9) |
| 95% CI^d^ | 6.6, 15.2 | 32.7, 46.1 | 9.4, 19.1 | | 17.2, 28.9 | 6.1, 15.1 | | 25.4, 39.1 |
| Abbreviations: CI = confidence interval; GMEU = geometric mean ELISA units; GMFR = geometric mean fold rise; GMT = geometric mean titer; ID_50_ = inhibitory dilution with a 50% concentration; Max = maximum; Min = minimum; N = number of participants in the assay-specific analysis Set; n1 = number of participants within each visit with non-missing data; n2 = number of participants with non-missing data at both visits of interest; n3 = number of participants who reported ≥4-fold increase with percentages calculated based on n2 as the denominator; LLOQ = lower limit of quantitation; SRR = seroresponse rate.  ^a^Baseline was defined as the last non-missing assessment prior to first vaccination. ^b^The 95% CI for GMT and GMFR were calculated based on the t-distribution of the log-transformed values then back transformed to the original scale for presentation. ^c^The SRR was defined as percentage of participants at each post vaccination visit with a titer ≥4-fold rise in MN_50_ or ID_50_ level. ^d^The 95% CI for SRR was calculated using the exact Clopper-Pearson method. ^e^The 95% CI for GMEU and GMFR were calculated based on the t-distribution of the log-transformed values then back transformed to the original scale for presentation. ^f^The SRR was defined as percentage of participants at each post vaccination visit with a titer ≥4-fold rise in anti-S IgG antibody level.  Note: Values less than LLOQ were replaced by 0.5 × LLOQ | | | | | | | | |
